# Metagenomic analysis reveals distinct patterns of gut lactobacillus prevalence, abundance, and geographical variation in health and disease

**DOI:** 10.1080/19490976.2020.1822729

**Published:** 2020-09-28

**Authors:** Tarini Shankar Ghosh, Jerome Arnoux, Paul W. O’Toole

**Affiliations:** a Department: School of Microbiology and APC Microbiome Ireland Institution, University College Cork, Cork, Ireland; b Department: UFR des Sciences et Techniques Institution, Université De Rouen, Normandie, France

**Keywords:** Gut microbiome, lactobacillus, regional variations, age variations, probiotic

## Abstract

Lactobacilli are exploited extensively for food fermentation and biotechnology. Some food and gut isolates have been developed as probiotics, for which species that may be commensal to the human host are considered desirable. However, the robustness of defining original niches for lactobacilli – food, environment, the gut – is questionable, and culture-independent analyses of prevalence in different human populations is lacking. Here we analyzed the abundance of lactobacilli in 6,154 subjects from a database of highly curated fecal shotgun metagenomics data spanning 25 nationalities, with ages ranging from infancy to 102 years. Twenty-five species were detected, which we assigned into low, medium, and high prevalence groups. The microbiome of apparently healthy individuals could be categorized into 6 clusters or Lactobacillotypes (LbTypes), with three of the Lbtypes being dominated by *L.* *delbrueckii*, *L.* *ruminis*, *L.* *casei*, and the other three comprising a combination of different species. These Lactobacillus clusters exhibit distinct global abundance patterns. The cluster prevalences also display distinct age-specific trends influenced by geography, with overall lactobacillus prevalence increasing significantly with age in North America and Europe but declining with age in non-Westernized societies. Regression analysis stratified by regional location identified distinct associations of the Lactobacillotypes with age, BMI, and gender. Cirrhosis, fatty-liver, , IBD and T2D were characterized by net gain of lactobacilli, whereas hypertension patients harbored depleted lactobacillus levels. Collectively these data indicate that the species abundance of gut lactobacilli is moderated by geography, diet, and interaction with the whole microbiome, and has strong interactions with diseases associated with a western lifestyle.

## Introduction

Humans, mammals, insects and plants harbor distinct communities of microorganisms with whom they have co-evolved.^1–3^ Despite the challenges of defining the “normal” or health-associated state of the microbiome, ^4^ there is emerging consensus that alterations in the composition and function of the animal gut microbiome are associated with pathophysiological syndromes or disease, both intestinal and extraintestinal.^5^ The precise molecular mechanisms whereby gut microbes could be involved in disease are still largely unexplained, but they include effects on metabolism, ^6^ immunity/inflammation, ^7^ tumorigenesis, ^8^ and signaling.^9^ Analysis of the microbiome state may also be informative for assessing risk of, diagnosing or managing disease. ^10–13^

The genus Lactobacillus encompasses an unusually diverse number of species that share the property of being found in nutrient-rich environments.^14^ Lactobacilli have been exploited extensively for food preservation, ^15^ for biotechnological applications, ^16^ and as health-promoting “probiotics”.^17^ The phenotypic diversity of the genus Lactobacillus is reflected in extraordinarily high genomic diversity, approaching that of other bacterial families. ^18,19^ The isolation sources for most lactobacilli may be broadly categorized as humans, animals, plants, food and environment, and major “lifestyle” assignment groupings coincide remarkably with phylogenomic clades, ^20^ indicating concerted evolution for niche adaptation.

Specialization of some lactobacillus species toward the human gut could indicate a commensal role, so it has historically been of interest to identify such species. Culture from human postmortem intestinal biopsies identified *L. gasseri, L. reuteri, L. salivarius, L. casei, L. plantarum* and *L. buchneri* as the most common lactobacillus species, ^21^ and lactobacilli were considered until relatively recently to be dominant taxa in the normal microbiota that reached greatest numbers in the small bowel. ^20^ However, data from an early tranche of molecular studies reviewed by Walter, ^22^ revealed low abundance levels (i.e., less than 1.0% total abundance) of sequences related to lactobacilli in/on fecal material or intestinal biopsies. The species most commonly found in the human gut, in addition to those listed above from the review by Reuter, ^21^ include *L. acidophilus, L. crispatus, L. johnsonii, L. ruminis, L. casei*/*paracasei, L. rhamnosus, L. plantarum, L. fermentum, L. brevis, L. delbrueckii, L. sakei, L. vaginalis*, and *L. curvatus*. ^22^ Identifying which lactobacilli are autochthonous to the human gut (formed where found) has also been of interest for the development of probiotics, based on an early misconception that species commonly used as probiotics must necessarily be able to transit the intestinal tract and then colonize the gut. ^23^ In reality, commensalism or autochthony is not an *a priori* requirement for an ingested microbe to have a beneficial effect on the host, and because so many lactobacillus species are naturally found in raw or fermented foods, isolating a given species from human stool does not reliably guarantee that species is autochthonous. Finally, many of the older culture-based literature relied on ambiguous phenotypic traits or since-altered species assignments, ^24^ making it hard to combine published datasets reliably to generate larger subject numbers for determining prevalence.

When administered as probiotics to humans, selected species and strains of lactobacilli have conferred benefits including alleviation of infant colic, ^25^ amelioration of lactose intolerance^26^ and reduced symptoms of atopic dermatitis. ^27^ Many lactobacilli are present in fermented foods that themselves have reported health benefits. ^28,29^ However, although human gut lactobacilli are generally considered beneficial, there are some reports of elevated lactobacillus abundance in the microbiome of people with some diseases e.g. Type 2 Diabetes, ^30^ obesity, ^31^ liver cirrhosis^32^ and even systemic autoimmunity. ^33^ However, these findings are controversial and may indicate association with disease symptoms rather than causation, because other studies have even suggested beneficial effects in these diseases. ^34–38^ Lactobacilli occasionally cause bacteremia or sepsis, almost always in immunocompromised patients, ^39^ and sometimes with strains administered as probiotics rather that strains already present in the subject. ^40,41^

The overall composition and function of the gut microbiome is influenced by external factors including geographical region, ^42^ ethnicity, ^43^ and diet. ^44,45^ How these factors intersect to modulate the lactobacillus species that are prevalent or abundant on a global basis is currently unknown. The availability of a large number of metagenomic datasets from globally distributed cohorts, apparently healthy controls and case studies, has allowed us to dissect the interaction of location, age, health, and disease with the abundance of commensal lactobacilli. We observe that the Lactobacillus composition in gut microbiomes displays distinct associations with geographical location, age, BMI, and gender of the individuals. Lactobacillus-microbiome configurations in western countries may represent recent reconfiguration of a primordial intestinal ecotype, among which a specific configuration of lactobacilli is positively associated with not only age and BMI, but also with multiple diseases and disease marker taxa.

## Results

### Lactobacillus prevalence in the human gut and Lactobacillus-specific microbiome configurations

Lactobacilli present in human stool may be either autochthonous species being shed, or allochthonous strains transiently acquired from food or the physical environment. We reasoned that transient carriage would be less of a factor in a very large dataset derived by culture-independent methods. The curatedMetagenomicData repository provides such a resource, comprising more than 5,700 fecal shotgun metagenome datasets from 34 studies. Of these, 21 are disease-microbiome studies, with 17 containing paired control and diseased samples^46^ (detailed in Supplementary Table S1). The remaining 13 study cohorts included only apparently healthy individuals, either from specific nationalities/ethnicities or age-groups. These datasets have all been collated and analyzed in a uniform manner which virtually eliminates bioinformatic-analysis-related variations across studies. We supplemented this dataset with 408 fecal microbiome profiles from our own ELDERMET project and a recently published case-control dataset comprising of IBD patients. ^47,48^

We first explored the prevalence of various Lactobacillus species across the 6,155 collated fecal microbiome datasets. Overall, 2141 of the 6155 samples harbored at least one Lactobacillus species, detected with a relative abundance of 0.01% (See Methods for the selection of this threshold of detection). We next used a linear regression-based strategy that quantified the association of various host-associated demographic factors with the Lactobacillus detection rate (that is the number of Lactobacillus species detected per sample) after taking into account the study-specific (technical) variations (by especially taking the study name as a confounder) (Supplementary Table S2a). The detection rates were significantly associated with geography (country), age-group and the study-conditions of the individuals. Study condition refers to the clinical status of the individual from whom the corresponding gut microbiome sample was collected (as part of the original study and then collated in the curatedMetagenomicData repository). Study condition indicates whether an individual is an apparently healthy control, or is suffering from a specific disease or has undergone a specific treatment like antibiotics or fecal microbiome transplantation (FMT). Thus, the above result indicates that even after adjusting for study-specific factors, the overall prevalence rates of Lactobacilli showed significant variation, not only with respect to the country or the age-group of the individuals, but also with clinical status. Furthermore, using PERMANOVA analysis, we observed that these associations remain significant even at the level of the abundance of the individual species, after accounting for the study-specific technical factors like DNA extraction method, sequencing depth, and sequencing methodology (Supplementary Table S2b). Next, we focussed only on the subset of 4,303 non-diseased controls to investigate whether apparently heathy individuals were characterized by distinct configurations of gut Lactobacilli and whether (and how) these configurations varied with respect to the geography, age-group and other demographic factors.

**Figure 1.**
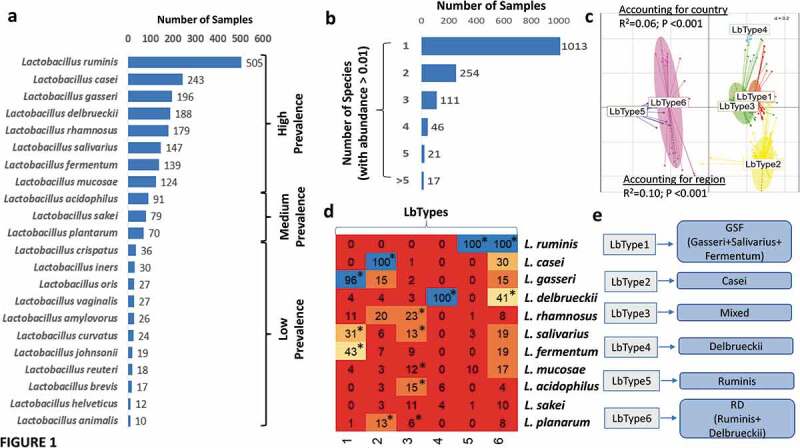
The gut microbiome of apparently healthy individuals is characterized by distinct lactobacillus populations. a. Bar plots showing the number of times each Lactobacillus species was detected (with abundance > 0.01%) in samples from ‘non-diseased’ control individuals. The species belonging to the ‘High-Prevalence’, ‘Medium-Prevalence’ and ‘Low-Prevalence’ groups of lactobacilli are demarcated. b. Bar showing the number of control individuals having different number of *Lactobacillus* species in their gut microbiomes. c. Principal Component Analysis (PCoA) plots (with the top 2 PCoA axes) showing the samples belonging to the different Lactobacillotypes (LbType). R-Squared and *P*-values of the PERMANOVA analysis testing the significance of these splits after considering both the country and the region as confounders is indicated. d. Heatmap on the left panel shows the percentage occurrence of each High and Medium prevalent *Lactobacillus* species in the six LbType. The percentage occurrence is the calculated from the number of times a given species was detected in a sample belonging to the given LbType divided by the total number of samples belonging to the LbType. Species that are significantly enriched in certain LbType were identified using Fishers’ Exact Test (estimate > 1 and *p*-value < 0.05) are indicated with *. e. Based on the significant enrichment patterns, the six LbTypes were named as described based on the dominant species in each cluster.

Overall, 1,459 of the 4,303 (34%) of ‘non-diseased’ controls harbored at least one Lactobacillus species, (Supplementary Table S3). The detection pattern encompassed 47 Lactobacillus species, with 22 of these (hereafter referred to as ‘rare’ lactobacilli) detected in less than 5 of the 1,459 samples. We detected 25 lactobacillus species above this threshold in 1,459 samples belonging to 31 cohorts from 22 countries (Figure 1(a)), with aggregate presence values ranging from 505 samples (*L. ruminis*), through 124 samples (*L. mucosae*), to 30 samples (*L. iners*) and below. For descriptive purposes, we divided the detected species into high (detected in greater than 100 samples), medium (detected in 50 to 100 samples) and low (less than 50 samples) prevalence groups (Figure 1(a)). The high-prevalence group included two species commonly consumed as probiotics, *L. casei* and *L. rhamnosus*, but the most prevalent species was *L. ruminis* which is also found in animals, and has the property of some strains being motile. ^49^ The species commonly used in combination with *Streptococcus thermophilus* for yogurt fermentation, *L. delbrueckii*, was the fourth most prevalent species (Figure 1(a)). The medium prevalence lactobacilli comprised three species found in fermented foods, *L. acidophilus* (also used as a probiotic), *L. sakei*, and *L. plantarum*. The low prevalence lactobacillus group included three species commonly found in the vagina (*L. iners, L. vaginalis*, and *L. jensenii*), the others being primarily food or animal associated species (Figure 1(a)). We also observed that 1013 of the 1459 samples were characterized by the presence of a single *Lactobacillus* spp. (69%), with only 6% having three or more species (Figure 1(b)).

To further clarify the lactobacillus configurations in the gut microbiomes of apparently healthy individuals, we asked if the individuals could be clustered in terms of their relatedness based on lactobacillus species abundance. This approach is conceptually similar to enterotypes^50^ but based on lactobacillus abundance profiles. This primarily consisted of two steps, the first being the identification of an optimal number of clusters and the second being identification of the key species associated with each cluster. For identification of the optimal number (k) of clusters, we adopted an iterative approach, wherein we performed 100 iterations. In each iteration, we randomly selected 50% of the samples and computed the silhouette scores for different cluster numbers (ranging from 2 to 20). The distribution of the silhouette scores across the 150 iterations for each cluster number is shown in Supplementary Figure S1a. The highest median silhouette scores were obtained when the clusters were k = 5 or 6. However, for k = 6, the variations across iterations was noticeably lower, indicating that the microbiomes could be clustered optimally into six clusters that are stable to variations across iterations. This identified the optimal number of clusters as six (Supplementary Figure S1b). We labeled these microbiome clusters as “Lactobacilllotypes” (or “LbTypes”) (Figure 1(c)). We further tested the robustness of these Lactobacillotype groupings using PERMANOVA analysis and Random Forest models. Regional factors capture variations in ethnicity, diet, lifestyle, and other socio-economic status, and have been shown in previous studies to have to have the strongest effect on the microbiome variations. ^43,48^ First, we observed that the variations in the compositional abundance of Lactobacilli across the different Lactobacillotypes remained significant after accounting for country of origin (R-squared = 0.06 and *P* < .001), continental region (R-squared = 0.10 and *P* < .001). The associations remained significant even after accounting study name as a confounder (PERMANOVA R-squared = 0.07 and *P* < .001), thus indicating that study-specific technical variations do not influence these associations).

We next validated the LbTypes using 100 iterations of Random Forest models, wherein for each iteration, we randomly selected 50% of the 1,462 samples to act as the training subsets to predict the Lactobacillotypes based on the lactobacillus profiles and subsequently tested this, trained on the remaining 50% of the samples. The models achieved an overall prediction accuracy of 98.4% across all LbTypes (Supplementary Figure S2a). The group-specific accuracies indicated similar levels of cluster granularities for almost all the LbTypes (accuracies of greater than 97%), except for LbType 6 (having the lowest group-specific accuracy at 93.5%).

Random Forest models provide the importance scores quantifying the predictive power of each feature (in this the case the lactobacillus abundance) in the LbType classification scheme. These species-specific feature importance scores indicated four species, *L. ruminis, L. gasseri, L. casei, L. delbrueckii*, as the top predictors of the Lactobacillotype classifications (Supplementary Figure S2b). These can be regarded as the signature taxa for each LbType. Three Lactobacillotypes (LbType 2, LbType 4 and LbType 5) were associated with each of the top predictor Lactobacillus species identified using the Random Forest models (with each signature species being present in at l00 of all samples in the corresponding LbTypes. To probe this further, we then identified the Lactobacilli significantly enriched in each of the Lactobacillotypes using Fishers’ exact test (Figure 1(d)). This combination of Random Forest models and Fishers’ exact tests enabled a clear identification of LbType-specific signatures. While LbType 1 was observed to be mixed, enriched with *L. gasseri* (present in 96% of the samples) (along with *L. salivarius* and *L. fermentum* present in 43% and 31% of the samples belonging to this LbType, respectively), LbTypes 2, 4 and 5 were each linked to *L. casei, L. delbrueckii*, and *L. ruminis*, respectively (each characteristic species being present in 100% of the samples belong to the corresponding LbType) (Figure 1(d)). Consequently, the LbTypes 1, 2, 4 and 5 were respectively named as Gasseri/Salivarius/Fermentum (or GSF), Casei, Delbrueckii, and Ruminis (Figure 1(e)). There were two other LbTypes (LbType 3 and LbType 6). LbType6 was enriched with *L. ruminis* (present in all the samples belonging to this LbType, similar to LbType 5) but additionally associated with *L. delbrueckii* (present in 41% of the samples) (Figure 1(d)). Because of this reason, it was named as Ruminis/Delbrueckii (or RD) (Figure 1(e)) In contrast to other LbTypes, we could not detect any signature species for LbType 3. This LbType was characterized by multiple species (none of which were detected in more than 25% of the samples. These included *L. rhamnosus* (present in 23% of the samples), followed by *L. salivarius, L. acidophilus, L. mucosae, L. sakei* (all present between 10 and 15% of the samples belonging to this LbType) as well as *L. fermentum* (which was present in 9% of this LbTypes’ samples). Consequently, LbType 3 was named as ‘Mixed’ (Figure 1(e)). (Hereafter these notations are used to refer to the LbTypes). The two *L. ruminis*-associated LbTypes were least related to the other LbTypes (Figure 1(c)).

We next investigated the association of these LbTypes with the global enterotypes (which we defined in a similar manner as the LbTypes but considering the global composition profiles of all the core taxa) across the 1,462 gut microbiome samples. All non-diseased individuals could be divided into three optimal clusters referred to as Enterotypes (or EnTypes 1–3) (Supplementary Figure S3a-c) associated with Prevotella, Bacteroides, and Bifidobacterium abundance, respectively (Supplementary Figure S3d; Supplementary Table S4). Using a combination of Fishers’ exact test and logistic regression models (See Methods and Supplementary Table S5), we observed associations of certain Enterotypes with different regions and age-groups. These reflected previously reported associations of particular microbial taxa with different regions and age-groups. The associations reflected the previous known associations of specific gut microbial members with age and geography. ^51,52^ These included significant enrichment of the Bifidobacterium-associated Enterotype 1 in infants; the enrichment of the Prevotella-associated Enterotype 3 in the Other (Non-industrialized) geography as well as the opposite trend observed for the Bacteroides-linked Enterotype 2. (Supplementary Figure S3d).

Analyzing the Lb-Type associations with the Enterotypes revealed a positive association between the ruminis-associated LbTypes 5 and 6 with the Prevotella-associated Enterotype 3 (Benjamini Hochberg FDR of Fishers’ Exact Test < 0.05; Supplementary Figure S3e). A similar link was also found between the LbTypes 1 (GSF), 2 (Casei) and 3 (Mixed), and the Bifidobacterium-associated Enterotype 1 (Benjamini Hochberg FDR of Fishers’ Exact Test < 0.05). The Bacteroides-associated Enterotype 2 was present across all LbTypes. However, significant positive associations were observed with LbTypes 3 (Mixed), 4 (Delbrueckii) and 5 (Ruminis) (Supplementary Figure S3e).

### Discrete patterns of Lactobacillus prevalence by region and country

As described above, geographic variation incorporates the key variations in ethnicity, diet, and life-styles, which have previously been identified as strong covariates of microbiome composition. ^43,44,48,52^ However, previous studies of lactobacillus abundance in the human gut did not overtly consider the effects of geographical region and country. The prevalence rates of Lactobacilli across the different regions displayed significant variations even after adjusting for study-specific effects (Supplementary Figure S4), with the nationalities belonging to the other non-industrialized regions having significantly higher prevalence rates as compared to North America (significantly lower rates of Lactobacilli prevalence).

**Figure 2.**
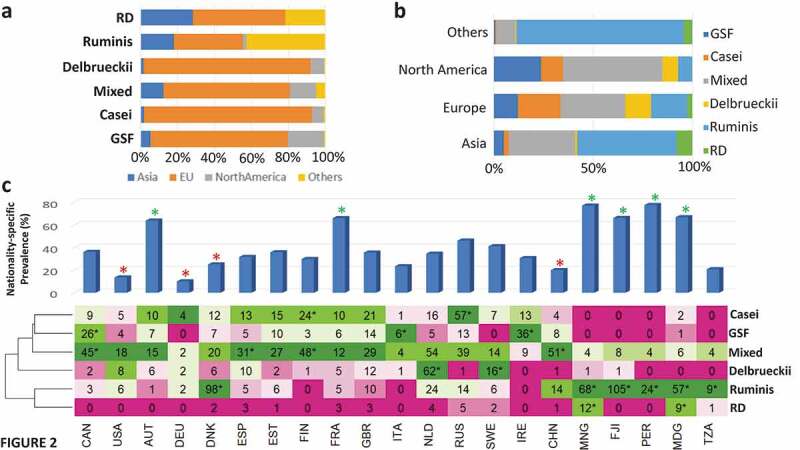
Lactobacillus species abundance in the gut displays significant region-specific trends. a. Stacked bar plots showing the region-wise percentage composition of each LbType b. Stacked bar plots showing the LbType percentage composition of each region. **c**. Top panel: Bar plots show the country-specific prevalence rates of Lactobacilli (percentage of samples from each country with at least one Lactobacillus species detected at abundance > 0.01%). Asterisks * in green indicate countries with significantly high prevalence rate, * in red indicate countries with significantly low prevalence rates (as compared to all others) (all with Benjamini-Hochberg FDR < 0.1). This was identified using Fishers’ exact tests (See Methods). Bottom panel shows the heatmap showing the number of times each LbType was detected across each country. * indicates significant enrichment of a LbType in a given country using Fishers’ exact test approach with Benjamini-Hochberg FDR < 0.1 (See Methods). For a given country, the colors of the cells are assigned based on the ranked detection of a LbType in that country (green for the highest detected LbType and red for the lowest detected LbType). * indicates significant enrichment of a LbType in a given country using Fishers’ exact test approach with Benjamini-Hochberg FDR < 0.1. For a given country, the colors of the cells were assigned based on the ranked detection of a LbType in that country (green for the highest detected LbType and red for the lowest detected LbType).

We determined the proportion of individuals whose microbiomes could be assigned to each LbType as a function of geographical region (Figure 2(a)) and the proportional representation with each region of the LbTypes (Figure 2(b)). The European subjects were characterized by a significant enrichment of LbTypes Delbrueckii, Casei, Mixed, and Gasseri/Salivarius/Fermentum (GSF) (Fishers’ Exact Test Benjamini-Hochberg FDR 2.14e-2, 3.4e-22, 5.63e-2 and 1.04e-2, respectively), and a significant lower prevalence of the two ruminis-associated LbTypes (Fishers’ Exact Test Benjamini-Hochberg FDR for Ruminis: 9.87e-6 and RD: 5.6e-2). In contrast, the *L. ruminis*-associated LbTypes were enriched in Asian subjects (Fishers’ Exact Test Benjamini-Hochberg FDR for Ruminis < 7.53e-7 and RD: 3.12e-3) and subjects belonging to the non-industrialized nationalities of Fiji, Peru, Tanzania and Madagascar (Fishers’ Exact Test Benjamini-Hochberg FDR for Ruminis: *P* < 5.11e-75 and RD: 0.32).

European and North American subjects had more even distribution of a greater number of LbTypes and both harbored the Delbrueckii and Casei LbTypes which was noticeably absent in Asians and in the subjects from non-industrialized regions (Figure 2(a)). Over thirty percent of North American subjects were of the mixed LbType (Figure 2(b)). The North American subjects also displayed the highest prevalence of the Gasseri/Fermentum/Salivarius (GSF) LbType. Compared to other regions and subjects, the European subjects displayed the highest prevalence of the Delbrueckii and Casei LbType which may relate to yogurt and probiotic consumption, although paired dietary intake data for the study subjects was not available.

The highest prevalence rates by country (Figure 2(c)) were found in Austria, France, and in three non-Industrialized countries, Mongolia, Fiji and Peru. In France, the mixed and Casei LbTypes were the most prevalent. In Austria, the most prevalent LbType was Delbrueckii. In other European countries, there were clear and sometimes surprising differences; Danish subjects were dominated by the Ruminis LbType whereas in Finland and Estonia, Casei was more dominant. Spain showed a similar profile of Lactobacillotype abundance as France, but Great Britain differed due to higher relative abundance of the Gasseri/Salivarius/Fermentum Lactobacillotype. In the non-industrialized regions of Mongolia, Fiji, Tanzania, Madagascar, and Peru, the Ruminis LbType was significantly dominant. The clear influence of geography on the Lactobacillus composition of the gut microbiome was observed with respect to both the LbType to country associations (Chi-Square Test *P* < 2.2e-16) as well as the variation of the species profiles across different countries (after adjusting for Study-specific effects) (PERMANOVA analysis of Species Composition Variance based on Kendall distances: *P* < .001).

These country-to-LbType relationships were also reflected in the country-to-species prevalence patterns (Supplementary Figure S5), whereby the Lactobacillus spcies composition of the subjects belonging to the North American and European countries were diverse (and characterized by distinctly lower prevalence of *L. ruminis*). In contrast, the subjects living in the non-industrialized countries were notably similar, being characterized by the dominance of *L. ruminis*.

### Region-specific association of Lactobacilli with age, Body Mass Index (BMI) and gender

Given that geography was significantly associated with both the prevalence of *Lactobacillus* species and LbTypes, we performed a region-stratified analysis of the association of host anthropometric factors including age, BMI and gender on the Lactobacillus relative abundance in the gut microbiome.

**Figure 3.**
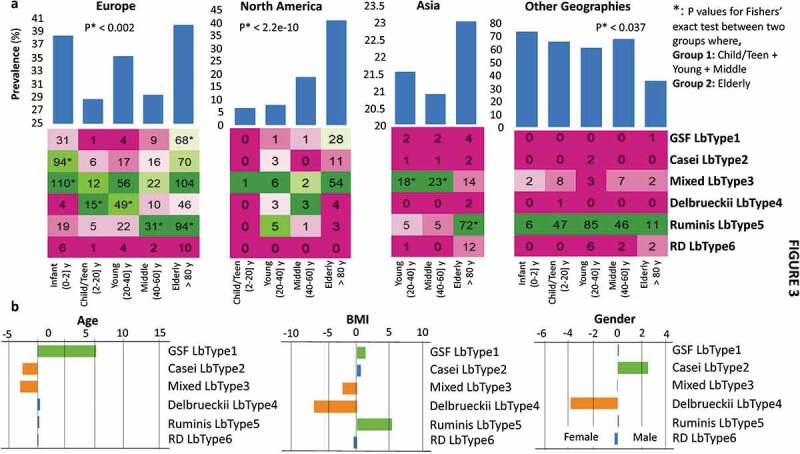
Host anthropometrics have specific associations with *Lactobacillus* species abundance, with age showing distinct region-dependent interaction. a. Bar plots on the top show the age-group specific prevalence rates of Lactobacilli across the four different regions. For a given region, the lactobacillus prevalence rates in a specified age-group were computed as the proportion of individuals (expressed as a percentage) in that age-group who have at least one lactobacillus species detected with abundance > 0.01. Fishers’ Exact Test *p*-values of the comparison of prevalence rates between Child/Young/Middle and the Elderly are shown for three regions of Europe, North America and the Other non-industrialized countries. Infants were not included in these groups because of the absence of infant samples from both North American and Asian regions, as well as the lack of consistent trends observed for the infant subjects across the other two regions. Heatmaps on the bottom panel show the number of times each LbType was detected in control individuals belonging to each age-group in each region. * indicates significant enrichment of a LbType in a given region. The age-groups of the individuals were defined as Infants: ≤ 2 years, Child/Teen: (2–20] years, Young: (20–40] years, Middle: (40–60] years and Elderly (> 60 years, with the maximum age being 102 years). b. Association extent computed using logistic regression (calculated by calculated by multiplying the -logarithm of *p*-values (of base 10) with the directionality of the association) considering the region as a confounder. Positive associations (with *P* < .05) are indicated in green. Negative associations (with *P* < .05) are indicated in orange. See Methods for details on the regression analysis.

Notwithstanding the fact that similarly-sized age groups were not equally distributed across all countries surveyed, in North America, the Lactobacillus prevalence rate in the Elderly being significantly higher than the Child/Teen/Young/Middle-aged groups (Figure 3(a); Fishers’ exact *P* < 2.2e-10). Similarly, for the European individuals, the Elderly were observed to have significantly higher Lactobacillus prevalence as compared to the Child/Teen, Young and Middle-aged groups (Fishers’ exact *P*-value < 0.002; Figure 3(a)). Only within Europe, the prevalence rates in the Infants were higher as compared to the Child/Teen/Young/Middle-aged groups (Fishers’ exact *P*-value < 0.013). While infant samples were not available from the North American and Asian regions, in the other non-industrialized regions, no significance difference in Lactobacillus prevalence rates was observed between the infants and those belonging to the Child/Teen/Young/Middle-aged groups. In the non-industrialized regions, however, we observed significantly lower Lactobacillus prevalence rates in the elderly (as compared to the Child/Teen/Young/Middle-aged groups). This was in contrast to that observed for the European and North American subjects. To further confirm that these observations were not consequences of country-specific biases in the proportional representation of age groups, we devised region-specific logistic regression models to compute the association of Lactobacillus prevalence rates with age after adjusting for country-specific variations (within each region) as confounders. The direction and the strength of the above associations were retained (however the negative association with age was not significant for the non-industrialized regions with *P* < .1) (Supplementary Table S7), confirming that Lactobacillus prevalence rates associate differentially with age depending upon the geographical location of the subject. Comparisons within the child/teen, young and middle were neither significant nor yielded any coherent patterns across regions.

We also detected distinct region-specific enrichments for certain LbTypes in specific age-groups. In North America, this was reflected in higher prevalence of the Mixed, Gasseri/Fermentum/Salivarius and the Casei LbTypes in the elderly, while European elderly subjects gained these three LbTypes plus the Ruminis LbType (Figure 3(a), lower). For the Asian cohort, age information was only available for the Chinese individuals. In this cohort, the gain of lactobacilli with age was less clear-cut and was not statistically significant, perhaps because the cohorts available featured very low numbers of elderly subjects. However, we detected significant differences in the LbType composition across age-groups, with the Young and the Middle age-groups being characterized by a significant enrichment of the mixed LbTypes, while the elderly were characterized by a significant gain of the Ruminis LbType. In the non-industrialized countries, an apparent decline in the Ruminis LbType prevalence was based on data for only 15 elderly subjects. Future analysis of more elderly subjects from these countries is required to investigate if our well-supported conclusion that Lactobacillus prevalence alters with age in North America and Europe also applies to those regions.

In addition to age, we also probed the association of Lactobacillus prevalence rates with BMI and gender separately within each geographical region. BMI data was available for 2092 subjects belonging to 16 studies (Supplementary Table S1). Increased lactobacillus abundance in obesity has been reported by one study, ^53^ but meta-analysis of the response of the gut microbiota to successful weight management interventions indicated that reduced lactobacillus abundance associated with weight loss. ^54^ We examined the prevalence of LbTypes as a function of Body Mass Index (BMI) in the gut metagenome data. For this we applied similar logistic regression models that checked the association of Lactobacilli prevalence first with BMI (and then with gender), while accounting for the country-specific variations as confounder (Supplementary Table S7). Region-stratified association analysis identified a marginally positive association between lactobacillus prevalence and BMI but only in European individuals (Logistic regression R = 0.024; *P* < .054) (Supplementary Table S7). This was not observed for any other geographical regions. However, there were variations in BMI ranges for the different studies with especially high BMI ranges for certain European cohorts like FengQ_2015 (median BMI = 27.6) and LeChatelierE_2013 (median BMI = 30.7) as compared to other regions. Such variations across region differences in BMI distributions may affect the ability to identify microbiome associations.

Further logistic regression analysis taking the region as cofounder identified *L. gasseri-salivarius-fermentum* LbType as positively associated with both age and BMI (Figure 3(b)). The mixed LbType showed the opposite trend. *L. delbrueckii* LbType was associated with lower BMI and the female gender. Interestingly, the *L. ruminis* LbType was positively associated with BMI. However, these results should be treated with caution as they could not be validated after taking into account the country-specific biases within each region. We could not perform this adjustment because of the gender/BMI biases within countries and the complete absence of certain LbType in different countries.

### Association between Lactobacillus abundance and disease

We next investigated the association of various Lactobacilli with different diseases. To avoid variations originating from differences in inclusion/exclusion criteria and experimental conditions, for each disease, we focussed only on cohorts specific to that disease. We identified 15 case-control studies corresponding to nine diseases that included at least 20 disease subjects and matched control samples (as part of the same study) (See Methods). We used logistic regression models to associate disease occurrence with the overall Lactobacillus prevalence rate as well as with the abundance of each *Lactobacillus* species after accounting for host anthropometric factors like age, BMI and gender.

**Figure 4.**
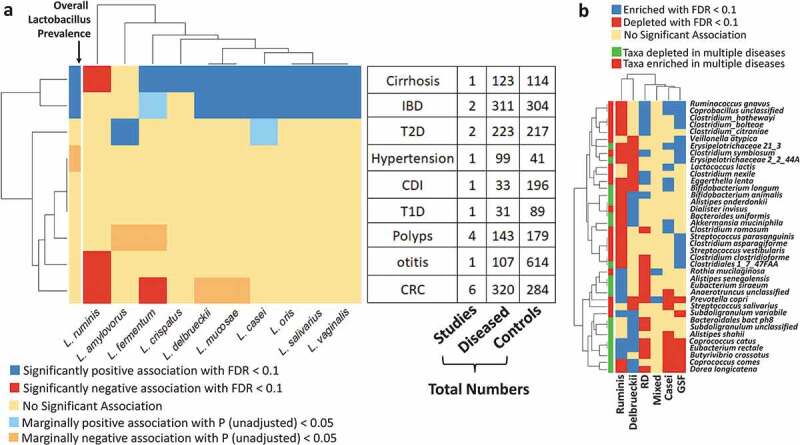
Lactobacillus species display distinct associations with multiple diseases and disease markers. a. Heatmap showing the significant association of the individual species as well as the overall *Lactobacillus* prevalence with the different diseases. The associations were obtained using Logistic regression models (within disease-specific country cohorts taking age, BMI and gender as confounders) (See Methods). Only those associations with either unadjusted *P* < .05 (shown as marginal associations) Benjamini-Hochberg FDR < 0.1 (as significant associations) are reported, along with the directionalities (as estimated from the regression models; that is positive or enriched if estimate > 0 and negative or depleted if estimate < 0). For each disease, we have shown the number of case-control studies included for each disease, the number of diseased subjects, and the number of control subjects. b. Heatmaps showing the association of the different generic disease markers with the different LbTypes identified using Mann-Whitney Tests (See Methods). Generic disease markers enriched or depleted in multiple diseases are indicated in green or red in the side color bars. Please refer to the original reference in Ghosh *et al*^48^ for details on the identification of these generic disease markers.

We identified 19 significant Lactobacillus-Disease associations (with Benjamini-Hochberg FDR < 0.1) (Figure 4(a)). These encompassed 11 Lactobacillus species covering six out of the nine diseases. Out of these six, IBD, Cirrhosis, and type-II diabetes (T2D) were observed to have a significant increase in the overall detection of Lactobacilli, with IBD and Cirrhosis associated with increased prevalence rates of six Lactobacillus species, namely, *L. gasseri, L. salivarius, L. mucosae, L. delbrueckii, L. vaginalis*, and *L. oris*. T2D, on the other hand, was associated with increased prevalence of *L. amylovorus*. Polyps (adenoma) and colorectal cancer (CRC) were associated with a decrease in the Lactobacillus prevalence rates. Notably, Ruminis was associated with a decreased prevalence in multiple diseases, including cirrhosis, CRC, and Otitis. These associations could either indicate a direct link, or an indirect effect whereby altered lactobacillus abundance is associated with changes in the overall microbiome composition, be they causative or consequential of the indicated pathophysiology.

We next investigated, in the non-diseased control individuals, if certain Lactobacillotypes were more associated with disease-like microbiome configurations as compared to others. For this purpose, we checked if any of the gut microbial taxa previously shown to be associated with multiple diseases also displayed significant variations across the different Lactobacillotypes. In a recent meta-analysis covering five major diseases (namely, colorectal cancer, inflammatory bowel disease, type II diabetes, polyps and cirrhosis), we identified a specific group of taxa that were associated (either enriched or depleted) across multiple diseases (as shown in Figure 4(b)). ^48^ In addition, we also observed that a subset of these taxa that were enriched in multiple diseases were also associated with frailty in the ELDERMET cohort. In this current analysis, we, therefore, checked for either enrichment or depletion of each of these multiple-disease-associated taxa in each of the six LbTypes. We found that subjects harboring the *L. gasseri-fermentum-salivarius* Lactobacillotype were enriched for several taxa including *C. citroniae, C. symbiosum, C. bolteae, C. asparagiforme, C. symbiosum, Clostridiales bacterium 1*_*47FAA*, that were not only enriched in multiple diseases but also associated with increased frailty in the elderly individuals (Figure 4(b)). ^48^ In contrast, this LbType was negatively associated with multiple health-associated taxa including *C. catus, E. rectale, B. crossotus, D. longicatena*. These data corroborate the association of *L. gasseri*, and *L. salivarius* with the altered microbiome found in multiple disease states (in other words as indicated by their increased detection in multiple diseases as seen in Figure 4(a)). In contrast, the *L. ruminis* Lactobacillotype showed the exact opposite trend, with enrichment of multiple health-associated taxa and depletion of the pathobionts that were enriched in subjects of the Gasseri/Fermentum/Salivarius LbType. The Delbrueckii and Casei LbTypes also showed multiple positive and negative associations with health-associated taxa, respectively.

## Discussion

Prior to the recent upsurge in culture-independent analyses of the gut microbiome, Lactobacilli were considered textbook examples of dominant or sub-dominant taxa. The vast number of 16S rRNA gene profiling studies and shotgun studies of the past decade showed this not to be true, but there had been no systematic studies of what Lactobacilli were present in what populations, and what, if any, host metadata co-varied with the Lactobacillus composition of the gut microbiome. Such insights could be helpful in the design and development of Lactobacillus-based (Lb-based) probiotic formulations. In spite of the availability of a multitude of over-the-counter Lb-based probiotic formulations, investigations into their clinical efficacy have yielded conflicting results, that are further confounded by the geographical region of the study-cohort, as well as by other host-associated factors like age (reviewed in^55^). Studies in animals and humans have indicated that the ability of an administered probiotic to engraft is dependent on predictive baseline host and microbiome features. ^56^ It is debatable whether or not probiotic engraftment is desirable or necessary, but the specific patterns of lactobacillus species-gut microbiome interactions we report here are consistent with the notion that an administered probiotic will show different rates of successful network interaction with different microbiome types. The efficacy of any species to ameliorate disease symptoms may thus be modulated by the diet, environment and the indigenous microbiome of the host. Thus, insights into the differential prevalence of Lactobacilli across the geographical and age-landscape may aid in the formulation of personalized or population-specific probiotic formulations.

In this context, a key challenge is the accurate species-level identification of the Lactobacilli in the gut microbiomes. Given their close phylogenetic relatedness, species assignment of lactobacilli is challenging based on 16S rRNA amplicon data (specifically those using short-read sequencing technologies) and so we relied on shotgun metagenomic sequencing data for the current study. The thresholds for the detection of each species has been validated for the Metaphlan software, ^57^ but our thresholds erred on the side of caution, so we acknowledge the possibility of under-detection of some species. Despite this technical caveat, the rank order of species prevalence aggregated across the subjects analyzed is broadly in line with older culture-based analyses (and reviewed in Introduction). We detected *L. buchneri* in only 9 samples above 0.01% abundance (Supplementary Table S1) although it was commonly cultured by Reuter, ^21^ which probably reflects the fact that it is isolated from pressed yeast, milk, cheese, and fermenting plant material and this could vary dramatically by time period and geography. *L. reuteri* was also detected at relatively low prevalence compared to historical culture-based data, being found in the current study in only 18 subjects above 0.01% relative abundance. Both *L. reuteri* and *L. buchneri* were sparsely detected in the Non-industrialized populations (0 for L. reuteri and 2 for L. buchneri) (Supplementary Figure S5). This is in line with studies by Walter that suggest human *L. reuteri* strains are a bottle-necked clonal line due to a relatively recent dramatic reduction in carriage of this species, ^22^ perhaps driven by industrialized Western diet.

The Enterotypes identified in the current study largely capture some of the known patterns of gut microbiome variation with age and region. The interesting insights obtained in the current study pertain to the specific LbType-Enterotype associations. *L. ruminis* is one of the few species suggested by Reuter to be autochthonous in humans which is consistent with our finding that it is by far the most highly prevalent species. ^21^ As well as being present in all major geographical regions that we could survey, this species was particularly prevalent and abundant in non-industrialized countries, where it correlated with the relative over-abundance Prevotella-associated enterotype 3. The genus *Prevotella* displays important strain-level diversity in phenotype not investigated here, ^58^ but the overall abundance is driven by fiber and complex carbohydrate intake, whereas *L. ruminis* strains found in humans, as well as being non-motile compared to animal isolates, have modest carbohydrate degradation capacity, ^59^ as do many lactobacillus species found in the human gut. ^60^ However, *L. ruminis* has been shown to effectively metabolize tetrasaccharides released from more complex substrates by gut microbiota taxa including *Coprococcus catus*. ^61^ This was further confirmed in the current study wherein we observed a significant enrichment of *C. catus* along with other fiber-degrading bacteria like *E. rectale, B. crossotus* in indivduals belonging to the *L. ruminis* LbType. Thus, the prevalence and abundance of *L. ruminis* in non-industrialized subjects could reflect its ability to cross-feed on substrates released from a high-fiber diet consumed by subjects in non-industrialized countries, who we recently showed have a very distinctive global microbiome compared to subjects in industrialized or Western countries. ^62^

The human gut microbiome changes with age, although age is a weaker co-variant with microbiome than region or country. ^48^ The age-dependent prevalence of lactobacillus is clearest in North America (increasing prevalence by age), which has the lowest fiber intake, whereas in the non-industrialized countries for which data was available the lactobacillus prevalence showed no noticeable difference across age into adulthood, but with a reduction in the elderly. This observation is largely based on the prevalence of the Ruminis and Ruminis/Delbrueckii (RD) LbTypes, and could also reflect diet, with declining fiber intake in the elderly. Similar trends of increasing prevalence with age are also reflected in Europe (from childhood till old age). However, a high prevalence in infancy could reflect probiotic consumption, especially of *L. casei* and *L. rhamnosus*. Previous studies investigating dietary differences have identified highest intake of dairy-based products in Europeans. ^63^

Our analyses of lactobacillus abundance interaction with disease does not allow distinction between cause and consequence. The enrichment of several lactobacillus species with cirrhosis, IBD, fatty liver disease and impaired glucose tolerance, viewed in isolation, present equivocal evidence for a role in disease. Based on our recent analysis of microbiome alterations in 2,500 case-control subjects, we studied lactobacillus associations with other genera and species that we showed displayed age-specific abundance changes in multiple diseases. ^48^ This revealed that the lactobacillus species that are higher or lower in abundance in the diseases tested show strong associations with the broader microbiome changes that are characteristic of these diseases, especially with the abundance of the microbiome signature taxa for these diseases. Thus, it is likely that the lactobacillus-disease associations are reflective of the overall gut ecological changes in these diseases. However, at least one medical case report exists where in injection of multiple Lb species including *L. salivarius* and *L. fermentum* was linked with primary biliary cirrhosis (in line with the results obtained in the current study albeit at the gut microbiome level). ^64^ Furthermore, these hitherto unreported associations between LbTypes and the known disease markers indicate the need to consider the baseline state of an individual’s microbiome in addition to other anthropometric factors prior to specific Lb-based probiotic administrations.

Different kinds of host-associated and technical factors are likely to affect the results of such meta-analyses that combine multiple datasets. We attempted to systematically account for a majority of these factors. First, we show that all the major host-associated factors (geography, age-group and disease) investigated in this study show significant associations with Lactobacillus detection rates even after adjusting for study-specific technical factors (the data for which was available for the curatedMetagenomicData repository). In the disease-association analyses, we further restricted our analyses to the study cohorts specific to each disease. This precludes biases in the results originating from varying inclusion/exclusion criteria as well as variations in experimental methodology. However, there were other factors which could not be accounted or investigated because of the nom-availability of these metadata in the curatedMetagenomicData repository. These include experimental/technical factors (like storage or transport of samples) as well as host-associated life-style/demographic data like polypharmacy information (which has a key influence on the composition of gut microbiome), ethnicity information pertaining to specific individuals belonging to a nationality as well as other information regarding dietary habits. Addition of these information in future versions of shotgun fecal microbiome data repositories could help shed light on the role of these factors on future microbiome-host association studies. Nevertheless, the information obtained herein provides direction for future studies that could focus on these factors in greater detail.

The practice of employing lactobacillus cultures for various beneficial functions and processes has been complicated by challenges in species identity, nomenclature and relatedness that will soon be simplified by the long-overdue taxonomic and phylogenetic overhaul of the 253 species. ^24,65^ Aided by the genome sequences of type species, and the ambition to sequence to sequence a million human gut microbiomes (“Million Microbiome of Humans Project” (https://en.mgitech.cn/news/114/)), we will soon have even greater geographic and age coverage to adjust for confounders in determining lactobacillus-microbiome-host interactions. However, the present study already shows that human gut lactobacilli reflect and respond to the geographic and lifestyle differences in the human host, and identify the key species involved.

## Materials and methods

### Collation of fecal microbiome datasets

The curatedMetagenomicData is available as a R package, downloadable from Bioconductor. The curatedMetagenomicData package (as on September 2019) was downloaded, filtered and processed as discussed in a previous study. ^48^ This created a repository of 5746 gut (fecal) microbiome profiles. To this, we added a further 189 and 219 from the ELDERMET cohort and a previous IBD case-control dataset. ^47,48^ The samples from the later two datasets were processed using the same approach as used for the samples in the curatedMetagenomicData repository, using Metaphlan2 and humann2. ^66,67^ The details of the datasets included in this study along with their geographical locations, the distribution availability of the age and BMI of the subjects, as well as the total number and the number of ‘control’ (apparently healthy) and ‘non-control’ (diseased and other conditions) have been listed in Supplementary Table S1. The details of the subject-specific metadata (including BMI, age, etc) for each study were already collected in the curatedMetagenomic repository from the individual studies (and used directly in this analysis). The collated fecal microbiome profiles corresponded to samples from 36 studies, spanning 25 nationalities across Europe, North America, Asia and Africa/South America/Oceania (grouped together as Others), distributed across five major age groups ranging from infancy to 102 years of age. The non-industrialized versus industrialized terminology was adopted from a recent study published recently by our group that used the same curatedMetagenomicData repository, ^62^ where in designations of industrialized and non-industrialized status were based on the classifications of the different nationalities by the United Nations Industrial Development Organization (UNIDO).

### Determination of Lactobacillus composition of the fecal microbiome

The first challenge here was to determine an appropriate abundance threshold to report a given *Lactobacillus* species as detected in a given metagenome. Using simulated metagenomes, previous studies on the validation of Metaphlan had observed that for abundance values greater than 0.01%, there was a linear relationship between the Metaphlan-calculated and the actual abundance values. ^57^ Therefore, we used this abundance threshold to identify a given Lactobacillus as being a present in each metagenome. Using this threshold, we identified the number of times a given Lactobacillus was detected across the different gut microbiome samples as well as the number of Lactobacilli detected in each sample. We specifically focussed on the non-diseased control individuals (tagged as ‘control’ in the study-condition metadata in the curatedMetagenomicData) for this purpose.

For obtaining a Lactobacillus specific species profile, we retained only those species belonging to the Lactobacillus genus and removed abundance values of less than 0.01%. For the overall microbiome profile, we restricted the species abundance profile to only those species that were present with abundance > 0.01% in 5% percent of the samples.

### Identification of Lactobacillotypes and Enterotypes and validation of Lactobacillotypes using Random Forest

Unlike previous approaches on Enterotyping that have either used Jensen-Shannon Divergence (JSD) or Drichlet Mixture Models (DMMs) on the global microbiome profiles, ^50,68^ in this study, we adopted a two-step strategy. First, we used a dimensionality reduction technique Principal Coordinate Analysis (PCoA) on the gut microbiome profile. The objective behind implementing a dimensionality reduction was reduction of the ‘noise’ associated with individual samples. Often, a group of samples, that may have an otherwise similar composition with respect to the large majority of the constituent taxa, may large variations amongst each other because of aberrantly high or low abundance of a few minority taxa that may have no contribution to the overall grouping schema. Utilizing a dimensionality-reduction technique and then representing each sample in terms of its top three axes (ordination axes explaining the largest variation) is expected to reduce this noise. Thus, in this study, we first performed a PCoA on the samples based on the intra-sample Spearman distances and subsequently represented the samples. We used the dudi.pco function (available in the ade4 package) and the cor function, both available in the R programming interface.

The next step was to identify an optimal number of clusters that achieved the best grouping of the samples. In this case, there are indices like silhouette scores or the Calinski-Harabasz (CH) index that can measure the clustering efficiency of a grouping based on ‘k’ clusters. Computing these scores for different values of ‘k’ and identifying the ‘k’ achieving the best efficiency was the approach that have been used in earlier studies on Enterotyping. ^50^ However, performing the step of identifying the optimal ‘k’ just once on the entire set of samples may not be appropriate, as it can also be biased by the presence of outliers in this specific set. A way of addressing would be to repeatedly subsample the set (taking a subset) and repeat the step of identifying the optimal ‘k’ for each iteration. The final ‘k’ would then be identified as the one that not only achieves a high clustering efficiency, but the clustering efficiency to repeated iterations. We adopted this approach, where in, we performed 150 iterations, and in each iteration, we computed the silhouette scores for ‘k’ = 2 to ‘k’ = 20. Clustering was performed using k-means using the Euclidean distances of the top three PCoA axes. The value of ‘k’ that passed the two criteria of high as well as stable clustering efficiency across the 150 iterations was identified as the optimal number of Lactobacillotypes or Enterotypes. These Lactobacillotypes and Enterotypes were further performed and validated visually using heatmaps (heatmap.2 function of the gplots package in R and the cutree function of the dendextend package) showing the clustering of the samples based on the Euclidean distances of the PCoA axes.

For the Lactobacillotypes, we performed an additional round of validation using Random Forest models wherein we again performed 100 iterations, where in each iteration, we trained models on 50% of the samples (for predicting the associated Lactobacillotype from the Lactobacilli composition) and tested the model on the rest 50%. The average classification accuracy per LbType as well as the full accuracy of classification was measured.

Associations between Enterotypes and the different regions and age-groups were performed using a combination of Fishers’ exact test and logistic regression models. While Fishers’ exact tests tested for association using simple count data, logistic regressions could test for the strength of these associations after taking into account the biases due to the various confounders (for example, biases in the representation of various age-groups across regions and biases in the representation of the different regions in sub-cohorts of individuals belonging to the different age-groups; described in detail in Supplementary Table S3).

### Profiling regional variations of the Lactobacillus populations in gut microbiome

The association of specific Lactobacillotypes with distinct regions or countries were tested using Fishers’ exact test. For each of these tests, a 2 × 2 contingency matrix was utilized which contained four values, namely the number of times a Lactobacillotype was detected in samples belonging to a given region/country, the remaining number of samples in the region/country, the number of times a Lactobacillotype was detected in all the other regions/countries, the remaining number of samples (that is those in which the Lactobacillotype is not detected) in all the other regions/countries. Fishers’ exact test provides two measures, namely the estimate (the extent of enrichment in the region/country versus the others, greater than 1 indicating enrichment and less than one indicating depletion) and the *p*-value (indicating the significance of association). For any region or country, enriched Lactobacillotypes were identified as those having estimates of greater than 1 and Benjamini–Hochberg FDR < 0.1.

For comparing the prevalence rates across countries, we used similar Fishers’ exact test-based approach, wherein we counted the total number of samples where in any lactobacillus was detected rather than a specific Lactobacillotype. Specifically, a 2 × 2 contingency matrix was utilized which contained four values, namely the number of times any Lactobacillus was detected in samples belonging to a given region/country, the remaining number of samples in the region/country, the number of times any Lactobacillus was detected in all the other regions/countries, the remaining number of samples (that is those in which Lactobacilli were not detected) in all the other regions/countries. For any region or country, enriched prevalence of lactobacilli was identified when estimates of greater than 1 were obtained along with Benjamini–Hochberg FDR < 0.1.

### Association analysis of Lactobacillotypes with host anthropometrics and disease

Association of *Lactobacillus* prevalence and Lactobacillotypes with host anthropometrics (age-group/age/BMI/gender) and disease were investigated using Fishers’ exact tests (as described above) and validated using Logistic regression models (that took into account various host-associated factors like region, country, age, BMI, and gender). The age-groups of the individuals were defined as Infants: ≤ 2 years, Child/Teen: (2–20] years, Young: (20–40] years, Middle: (40–60] years and Elderly (> 60 years, with the maximum age being 102 years). The selection of these host-associated factors as possible de-confounders was done based on the results of our previous meta-analysis, ^48^ wherein these host-associated metadata were present in more than 30% of the subjects, and also had the highest effect on the microbiome composition.

The region-stratified logistic regression models (separate models for each region) associating Lactobacillus prevalence with host anthropometrics were obtained as follows:

glm(LactobacillusDetected (1: Detected and 0: Not Detected) ~ Country + Age/BMI/Gender, family = ”binomial”)

These models considered the country-wise variations as a confounder.

The association of Lactobacillotypes to host anthropometrics were computed as:

glm(Lactobacillotype X (1: If sample belongs to LbType X and 0: If it does not belong to LbType X) ~ Region + Age/BMI/Gender, family = ”binomial”)

These models considered the region-wise variations as a confounder.

We next probed the association of the overall prevalence or the different Lactobacilli with disease. First, we removed those disease datasets from this analysis that contained fewer than 20 diseased subjects. This resulted in a list of 11 diseases, namely, adenoma (or polyps), atherosclerosis (AS), Clostridium difficile infection (CDI), cirrhosis, colorectal cancer (CRC), hypertension, inflammatory bowel disease (IBD), otitis, premature-born, type-I diabetes (T1D) and type-II diabetes (T2D). Out of these 11 diseases, for AS and premature born individuals, there were no matched controls sequenced as part of the same study (Supplementary Table S1), thereby restricting us to focus on the remaining nine diseases for this analysis. This included 15 matched case-control study datasets. For each disease, we constituted disease-specific bins by collating ‘control’ and ‘case’ samples from the ‘case-control’ study datasets corresponding to that disease.

Subsequently, only within the disease-specific bins, we utilized logistic regression models (as follows):

glm(LactobacillusDetected~Age+BMI+gender+DiseaseStatus, method = ”binomial”) (for overall prevalence)

glm(Lactobacillus Species ‘X’ Detected~Age+BMI+gender+DiseaseStatus, method = ”binomial”) (for overall prevalence) (for a specific Lactobacilli X)

The list of multiple disease markers were obtained from a previous study by our group. ^48^ The association of generic disease markers with Lactobacillotypes was performed using Mann-Whitney Tests. For each Lactobacillotype, the abundances of the disease markers were compared between all samples belonging to a Lactobacillotype and those not belonging to the Lactobacillotype. Species that were significantly enriched or depleted with Benjamini-Hochberg FDR < 0.1 were identified.

## Supplementary Material

Supplemental Material
