# Supplementary material for: Metagenomic analysis reveals distinct patterns of gut lactobacillus prevalence, abundance, and geographical variation in health and disease
Source: Gut Microbes. 2020 Sep 28;12(1):1822729. doi: 10.1080/19490976.2020.1822729 (PMC7524322; doi:10.1080/19490976.2020.1822729)
Supplement: Supplemental Material [file KGMI_A_1822729_SM9159.zip › Supplementary information/Revised2_SupplementaryTableS4.pdf]

Supplementary Table S4: Taxa differentially abundant in each of the Global Enterotypes (A) EnType1 (B) EnType2 and (C) EnType3. For each Enterotype, Mann-Whitney Tests were performed between the samples belonging to the given Enterotype and those belonging to the other two Enterotypes. Taxa showing significant differences in their abundances (with Benjamini-Hochberg FDR < 0.05) are reported for each along with their directionalities. The prevalence rates and the mean ranked abundance of each taxa within each Enterotype is also provided. Ranked abundances and Prevalence were obtained as indicated in the footnote of this table.

(A)

Species	EnteroType	Direction	FDR	Prevalence	Mean Ranked Abundance
Bifidobacterium longum	EnType1	ENRICHED	8.45E-05	79.30	0.58
Bifidobacterium bifidum	EnType1	ENRICHED	1.58E-12	50.39	0.48
Escherichia coli	EnType1	ENRICHED	1.23E-13	87.11	0.65
Bifidobacterium breve	EnType1	ENRICHED	4.04E-69	41.02	0.52
Bacteroides vulgatus	EnType1	ENRICHED	3.36E-17	53.91	0.27
Bacteroides ovatus	EnType1	ENRICHED	8.88E-26	37.89	0.21
Ruminococcus gnavus	EnType1	ENRICHED	2.27E-08	57.03	0.49
Prevotella bivia	EnType1	ENRICHED	4.42E-19	22.27	0.23
Bacteroides dorei	EnType1	ENRICHED	5.44E-16	34.38	0.23
Finegoldia magna	EnType1	ENRICHED	2.21E-51	29.30	0.30
Stenotrophomonas maltophilia	EnType1	ENRICHED	3.74E-29	17.58	0.18
Porphyromonas bennonis	EnType1	ENRICHED	2.22E-33	25.78	0.28
Enterococcus faecium	EnType1	ENRICHED	1.33E-16	26.17	0.27
Enterococcus faecalis	EnType1	ENRICHED	5.89E-70	48.44	0.50
Bacteroides caccae	EnType1	ENRICHED	5.37E-25	25.78	0.16
Bifidobacterium dentium	EnType1	ENRICHED	1.04E-05	35.94	0.37
Bifidobacterium pseudocatenulatum	EnType1	ENRICHED	1.19E-08	14.45	0.14
Afipia broomeae	EnType1	ENRICHED	2.15E-35	14.84	0.16
Lactobacillus casei paracasei	EnType1	ENRICHED	3.17E-11	32.03	0.34
Prevotella buccalis	EnType1	ENRICHED	1.29E-27	23.83	0.24
Veillonella ratti	EnType1	ENRICHED	2.63E-19	8.98	0.11
Parabacteroides distasonis	EnType1	ENRICHED	1.10E-26	21.48	0.16
Bacteroides thetaiotaomicron	EnType1	ENRICHED	4.31E-20	26.17	0.20
Gardnerella vaginalis	EnType1	ENRICHED	3.94E-11	13.67	0.17
Haemophilus parainfluenzae	EnType1	ENRICHED	3.22E-06	32.81	0.27
Enterococcus avium	EnType1	ENRICHED	9.22E-17	14.45	0.15
Sutterella wadsworthensis	EnType1	ENRICHED	8.34E-15	17.19	0.15
Bradyrhizobium sp DFCI 1	EnType1	ENRICHED	2.79E-17	8.98	0.09
Peptoniphilus lacrimalis	EnType1	ENRICHED	6.38E-28	19.53	0.20
Prevotella timonensis	EnType1	ENRICHED	2.89E-23	21.88	0.25
Campylobacter ureolyticus	EnType1	ENRICHED	5.44E-20	11.33	0.12
Lactobacillus rhamnosus	EnType1	ENRICHED	1.82E-61	41.02	0.44

Species	EnteroType	Direction	FDR	Prevalence	Mean Ranked Abundance
Eggerthella unclassified	EnType1	ENRICHED	1.92E-10	57.81	0.50
Parabacteroides unclassified	EnType1	ENRICHED	4.15E-07	20.70	0.16
Anaerococcus vaginalis	EnType1	ENRICHED	3.35E-35	24.22	0.25
Coprobacillus unclassified	EnType1	ENRICHED	2.18E-13	41.02	0.40
Campylobacter hominis	EnType1	ENRICHED	0.0024423	9.38	0.10
Porphyromonas asaccharolytica	EnType1	ENRICHED	7.62E-14	19.14	0.24
Bacteroides faecis	EnType1	ENRICHED	5.26E-24	5.08	0.07
Lactobacillus iners	EnType1	ENRICHED	1.22E-07	7.42	0.08
Parabacteroides goldsteinii	EnType1	ENRICHED	2.20E-07	3.13	0.04
Enterococcus durans	EnType1	ENRICHED	6.70E-06	10.16	0.11
Megasphaera micronuciformis	EnType1	ENRICHED	3.46E-06	12.50	0.15
Klebsiella unclassified	EnType1	ENRICHED	1.29E-19	28.13	0.33
Prevotella disiens	EnType1	ENRICHED	9.86E-05	14.45	0.16
Lactobacillus gasseri	EnType1	ENRICHED	5.44E-07	26.17	0.28
Cupriavidus unclassified	EnType1	ENRICHED	1.37E-24	13.28	0.14
Lactobacillus plantarum	EnType1	ENRICHED	0.0002426	11.72	0.15
Clostridium ramosum	EnType1	ENRICHED	5.09E-10	28.91	0.30
Anaerococcus prevotii	EnType1	ENRICHED	7.92E-40	17.19	0.18
Klebsiella oxytoca	EnType1	ENRICHED	3.10E-33	26.95	0.30
Bacteroides xylanisolvens	EnType1	ENRICHED	5.30E-43	12.50	0.09
Enterococcus casseliflavus	EnType1	ENRICHED	1.70E-23	13.67	0.16
Porphyromonas somerae	EnType1	ENRICHED	3.39E-06	10.16	0.12
Afipia unclassified	EnType1	ENRICHED	1.69E-36	15.23	0.16
Anaerococcus obesiensis	EnType1	ENRICHED	5.61E-36	20.70	0.21
Atopobium vaginae	EnType1	ENRICHED	8.97E-09	7.42	0.10
Peptoniphilus harei	EnType1	ENRICHED	4.69E-26	14.06	0.16
Lactobacillus brevis	EnType1	ENRICHED	0.0145177	3.91	0.05
Anaerococcus tetradius	EnType1	ENRICHED	7.14E-06	2.73	0.03
Anaerostipes hadrus	EnType1	ENRICHED	2.04E-50	11.72	0.09
Enterococcus gallinarum	EnType1	ENRICHED	5.95E-14	10.16	0.12
Porphyromonas uenonis	EnType1	ENRICHED	3.37E-06	11.72	0.13
Subdoligranulum sp 4 3 54A2FAA	EnType1	ENRICHED	0.000268	3.13	0.03
Lachnospiraceae bacterium 3 1 57FAA CT1	EnType1	ENRICHED	1.76E-02	12.89	0.14
Flavonifractor plautii	EnType1	ENRICHED	0.0250495	21.48	0.20
Mobiluncus curtisii	EnType1	ENRICHED	1.93E-22	11.33	0.12

Species	EnteroType	Direction	FDR	Prevalence	Mean Ranked Abundance
Staphylococcus epidermidis	EnType1	ENRICHED	2.18E-13	5.86	0.07
Rhodopseudomonas palustris	EnType1	ENRICHED	3.15E-21	10.94	0.11
Megasphaera elsdenii	EnType1	ENRICHED	0.0006497	4.69	0.05
Anaerococcus hydrogenalis	EnType1	ENRICHED	1.76E-17	5.86	0.08
Lactobacillus mucosae	EnType1	ENRICHED	0.0003807	2.34	0.03
Anaerococcus lactolyticus	EnType1	ENRICHED	1.67E-25	8.20	0.12
Varibaculum cambriense	EnType1	ENRICHED	7.45E-35	16.02	0.20
Stenotrophomonas unclassified	EnType1	ENRICHED	2.23E-19	8.20	0.10
Dorea unclassified	EnType1	ENRICHED	9.70E-09	17.97	0.21
Propionibacterium avidum	EnType1	ENRICHED	1.28E-08	3.52	0.04
Actinomyces urogenitalis	EnType1	ENRICHED	1.90E-14	7.03	0.07
Peptoniphilus duerdenii	EnType1	ENRICHED	1.31E-13	8.20	0.10
Clostridium asparagiforme	EnType1	ENRICHED	4.56E-05	9.38	0.10
Lactobacillus sakei	EnType1	ENRICHED	0.0023494	0.78	0.01
Anaerostipes unclassified	EnType1	ENRICHED	7.35E-07	10.55	0.12
Morganella morganii	EnType1	ENRICHED	0.0002703	5.86	0.06
Anaeroglobus geminatus	EnType1	ENRICHED	0.0035152	3.91	0.05
Prevotella bergensis	EnType1	ENRICHED	2.38E-17	7.42	0.09
Lachnospiraceae bacterium 1 4 56FAA	EnType1	ENRICHED	0.0011429	5.86	0.06
Mobiluncus unclassified	EnType1	ENRICHED	5.13E-05	3.52	0.05
Dialister micraerophilus	EnType1	ENRICHED	2.21E-14	6.25	0.08
Clostridium citroniae	EnType1	ENRICHED	1.08E-10	5.86	0.07
Clostridium difficile	EnType1	ENRICHED	2.57E-06	5.08	0.06
Streptococcus agalactiae	EnType1	ENRICHED	0.0310133	2.73	0.04
Corynebacterium pyruviciproducens	EnType1	ENRICHED	6.45E-10	3.52	0.04
Clostridiales bacterium BV3C26	EnType1	ENRICHED	1.61E-07	3.52	0.04
Streptococcus infantis	EnType1	ENRICHED	3.06E-08	3.91	0.08
Peptoniphilus timonensis	EnType1	ENRICHED	1.84E-09	3.91	0.05
Facklamia hominis	EnType1	ENRICHED	1.71E-08	4.30	0.05
Slackia unclassified	EnType1	ENRICHED	4.97E-05	3.91	0.06
Corynebacterium amycolatum	EnType1	ENRICHED	6.34E-10	5.86	0.07

Species	EnteroType	Direction	FDR	Prevalence	Mean Ranked Abundance
Erysipelotrichaceae bacterium 21 3	EnType1	ENRICHED	3.39E-07	7.03	0.11
Peptoniphilus rhinitidis	EnType1	ENRICHED	1.31E-12	3.13	0.06
Corynebacterium aurimucosum	EnType1	ENRICHED	0.0001332	2.73	0.03
Granulicatella unclassified	EnType1	ENRICHED	2.46E-06	9.38	0.15
Facklamia unclassified	EnType1	ENRICHED	0.0023396	2.73	0.04
Actinomyces europaeus	EnType1	ENRICHED	7.42E-05	1.56	0.02
Corynebacterium pseudogenitalium	EnType1	ENRICHED	0.010782	1.56	0.02
Streptococcus peroris	EnType1	ENRICHED	2.12E-17	7.81	0.14
Coriobacteriaceae bacterium BV3Ac1	EnType1	ENRICHED	1.27E-15	5.86	0.07
Staphylococcus aureus	EnType1	ENRICHED	1.55E-06	5.08	0.08
Comamonas unclassified	EnType1	ENRICHED	0.0439052	1.17	0.02
Actinomyces neuii	EnType1	ENRICHED	0.0012735	1.95	0.02
Ralstonia pickettii	EnType1	ENRICHED	0.000362	2.73	0.05
Corynebacterium tuberculostearicum	EnType1	ENRICHED	0.0001351	2.34	0.03
Facklamia ignava	EnType1	ENRICHED	0.0007598	1.56	0.02
Slackia exigua	EnType1	ENRICHED	0.0122685	1.95	0.02
Propionimicrobium lymphophilum	EnType1	ENRICHED	0.0001415	1.56	0.03
Acidaminococcus fermentans	EnType1	DEPLETED	0.0283901	1.56	0.02
Actinomyces graevenitzii	EnType1	DEPLETED	0.0012567	1.56	0.05
Actinomyces odontolyticus	EnType1	DEPLETED	2.11E-05	3.91	0.12
Actinomyces viscosus	EnType1	DEPLETED	0.0042318	1.17	0.02
Alistipes finegoldii	EnType1	DEPLETED	8.31E-38	12.11	0.09
Alistipes indistinctus	EnType1	DEPLETED	1.87E-30	2.73	0.02
Alistipes onderdonkii	EnType1	DEPLETED	1.17E-29	21.09	0.13
Alistipes putredinis	EnType1	DEPLETED	6.31E-55	14.84	0.06
Alistipes shahii	EnType1	DEPLETED	4.12E-68	8.98	0.05
Anaerotruncus colihominis	EnType1	DEPLETED	9.99E-12	3.91	0.04
Bacteroides cellulosilyticus	EnType1	DEPLETED	3.41E-25	5.86	0.05
Bacteroides clarus	EnType1	DEPLETED	1.50E-11	2.34	0.03
Bacteroides coprocola	EnType1	DEPLETED	8.16E-09	3.13	0.03
Bacteroides coprophilus	EnType1	DEPLETED	0.0074204	0.78	0.01

Species	EnteroType	Direction	FDR	Prevalence	Mean Ranked Abundance
Bacteroides eggerthii	EnType1	DEPLETED	1.92E-09	8.20	0.08
Bacteroides finegoldii	EnType1	DEPLETED	4.67E-13	3.52	0.03
Bacteroides intestinalis	EnType1	DEPLETED	5.25E-13	2.34	0.02
Bacteroides massiliensis	EnType1	DEPLETED	1.02E-13	11.33	0.09
Bacteroides nordii	EnType1	DEPLETED	1.41E-09	0.39	0.01
Bacteroides plebeius	EnType1	DEPLETED	2.16E-09	2.73	0.03
Bacteroides salyersiae	EnType1	DEPLETED	0.000256	2.73	0.03
Bacteroides stercoris	EnType1	DEPLETED	1.18E-21	16.02	0.13
Bacteroides uniformis	EnType1	DEPLETED	1.83E-22	51.95	0.23
Barnesiella intestinihominis	EnType1	DEPLETED	1.36E-49	10.55	0.06
Bifidobacterium adolescentis	EnType1	DEPLETED	5.42E-25	30.86	0.25
Bifidobacterium angulatum	EnType1	DEPLETED	3.32E-05	0.78	0.01
Bifidobacterium animalis	EnType1	DEPLETED	0.0043319	4.30	0.06
Bifidobacterium catenulatum	EnType1	DEPLETED	1.52E-15	7.03	0.08
Bilophila wadsworthia	EnType1	DEPLETED	5.97E-32	7.81	0.07
Blautia hydrogenotrophica	EnType1	DEPLETED	0.038117	2.73	0.03
Butyricimonas synergistica	EnType1	DEPLETED	2.29E-08	0.00	0.00
Butyrivibrio crossotus	EnType1	DEPLETED	8.26E-26	1.95	0.02
Catenibacterium mitsuokai	EnType1	DEPLETED	9.25E-26	2.73	0.02
Clostridium bartlettii	EnType1	DEPLETED	2.82E-20	25.39	0.19
Clostridium leptum	EnType1	DEPLETED	1.36E-38	7.03	0.06
Clostridium scindens	EnType1	DEPLETED	5.13E-10	0.39	0.01
Collinsella aerofaciens	EnType1	DEPLETED	2.34E-52	29.30	0.17
Coprococcus catus	EnType1	DEPLETED	1.91E-89	6.64	0.04
Coprococcus comes	EnType1	DEPLETED	9.23E-93	9.77	0.05
Coprococcus eutactus	EnType1	DEPLETED	2.96E-16	2.34	0.03
Desulfovibrio desulfuricans	EnType1	DEPLETED	1.06E-15	3.52	0.04
Desulfovibrio piger	EnType1	DEPLETED	1.52E-17	1.56	0.01
Dialister invisus	EnType1	DEPLETED	0.0002243	19.53	0.15
Dialister succinatiphilus	EnType1	DEPLETED	1.61E-07	0.39	0.01
Dorea formicigenerans	EnType1	DEPLETED	5.71E-99	11.72	0.06
Dorea longicatena	EnType1	DEPLETED	2.25E-86	19.14	0.09
Eubacterium biforme	EnType1	DEPLETED	4.14E-38	5.08	0.04
Eubacterium cylindroides	EnType1	DEPLETED	0.0001758	1.95	0.02

Species	EnteroType	Direction	FDR	Prevalence	Mean Ranked Abundance
Eubacterium eligens	EnType1	DEPLETED	5.15E-78	17.58	0.09
Eubacterium hallii	EnType1	DEPLETED	1.97E-94	12.89	0.06
Eubacterium ramulus	EnType1	DEPLETED	4.74E-79	4.69	0.03
Eubacterium rectale	EnType1	DEPLETED	1.29E-100	26.95	0.08
Eubacterium siraeum	EnType1	DEPLETED	4.07E-49	13.28	0.08
Eubacterium ventriosum	EnType1	DEPLETED	2.25E-58	2.73	0.01
Faecalibacterium prausnitzii	EnType1	DEPLETED	6.84E-98	46.88	0.10
Gemella sanguinis	EnType1	DEPLETED	0.0004995	1.17	0.03
Gordonibacter pamelaeae	EnType1	DEPLETED	1.20E-10	7.42	0.08
Holdemania filiformis	EnType1	DEPLETED	3.59E-28	3.52	0.04
Lactobacillus delbrueckii	EnType1	DEPLETED	9.99E-07	3.13	0.03
Lactobacillus ruminis	EnType1	DEPLETED	2.15E-22	6.64	0.06
Methanobrevibacter smithii	EnType1	DEPLETED	6.97E-28	7.03	0.06
Methanosphaera stadtmanae	EnType1	DEPLETED	3.35E-06	0.78	0.01
Mitsuokella multacida	EnType1	DEPLETED	2.41E-09	0.39	0.01
Odoribacter splanchnicus	EnType1	DEPLETED	3.16E-45	2.73	0.02
Oxalobacter formigenes	EnType1	DEPLETED	1.95E-22	0.00	0.00
Parabacteroides johnsonii	EnType1	DEPLETED	6.87E-12	1.95	0.02
Parabacteroides merdae	EnType1	DEPLETED	1.77E-33	20.70	0.13
Paraprevotella clara	EnType1	DEPLETED	2.26E-14	1.95	0.02
Paraprevotella xylaniphila	EnType1	DEPLETED	1.82E-14	0.78	0.01
Phascolarctobacterium succinatutens	EnType1	DEPLETED	4.71E-22	7.42	0.06
Prevotella copri	EnType1	DEPLETED	5.94E-37	13.28	0.12
Prevotella stercorea	EnType1	DEPLETED	4.90E-24	1.56	0.01
Pseudoflavonifractor capillosus	EnType1	DEPLETED	2.41E-24	0.00	0.01
Roseburia hominis	EnType1	DEPLETED	5.77E-87	10.16	0.05
Roseburia intestinalis	EnType1	DEPLETED	2.81E-52	18.75	0.13
Roseburia inulinivorans	EnType1	DEPLETED	1.56E-92	14.06	0.07
Rothia mucilaginosa	EnType1	DEPLETED	2.86E-11	9.77	0.13
Ruminococcus albus	EnType1	DEPLETED	7.07E-22	0.00	0.01
Ruminococcus bromii	EnType1	DEPLETED	3.51E-56	23.44	0.13
Ruminococcus callidus	EnType1	DEPLETED	6.94E-50	4.30	0.03
Ruminococcus champanellensis	EnType1	DEPLETED	0.0081949	0.78	0.01
Ruminococcus flavefaciens	EnType1	DEPLETED	1.46E-15	0.00	0.00

Species	EnteroType	Direction	FDR	Prevalence	Mean Ranked Abundance
Ruminococcus lactaris	EnType1	DEPLETED	2.09E-38	8.59	0.07
Ruminococcus obeum	EnType1	DEPLETED	4.13E-96	25.39	0.10
Ruminococcus torques	EnType1	DEPLETED	8.29E-43	66.02	0.24
Solobacterium moorei	EnType1	DEPLETED	0.0153823	5.47	0.08
Streptococcus australis	EnType1	DEPLETED	4.67E-15	7.42	0.09
Streptococcus gordonii	EnType1	DEPLETED	2.03E-10	2.34	0.04
Streptococcus infantarius	EnType1	DEPLETED	6.02E-03	2.73	0.04
Streptococcus mutans	EnType1	DEPLETED	0.0006435	3.52	0.05
Streptococcus parasanguinis	EnType1	DEPLETED	6.22E-09	40.23	0.34
Streptococcus salivarius	EnType1	DEPLETED	6.41E-38	38.67	0.26
Streptococcus sanguinis	EnType1	DEPLETED	1.41E-16	1.95	0.03
Streptococcus thermophilus	EnType1	DEPLETED	1.23E-10	21.09	0.18
Streptococcus vestibularis	EnType1	DEPLETED	7.00E-08	8.59	0.10
Subdoligranulum variabile	EnType1	DEPLETED	2.45E-36	0.00	0.01
Adlercreutzia equolifaciens	EnType1	DEPLETED	3.52E-35	4.30	0.03
Alistipes senegalensis	EnType1	DEPLETED	7.80E-52	2.73	0.02
Alistipes sp AP11	EnType1	DEPLETED	5.87E-11	0.78	0.01
Alloprevotella unclassified	EnType1	DEPLETED	0.0001345	1.17	0.02
Anaerotruncus unclassified	EnType1	DEPLETED	4.11E-52	2.73	0.02
Bacteroidales bacterium ph8	EnType1	DEPLETED	3.02E-49	5.86	0.03
Bilophila unclassified	EnType1	DEPLETED	6.52E-47	15.23	0.10
Brachyspira unclassified	EnType1	DEPLETED	0.0070072	0.00	0.00
Burkholderiales bacterium 1 1 47	EnType1	DEPLETED	6.49E-17	5.86	0.06
Butyrivibrio unclassified	EnType1	DEPLETED	2.47E-08	1.95	0.03
Candidatus Zinderia insecticola	EnType1	DEPLETED	0.0192743	0.00	0.01
Clostridiaceae bacterium JC118	EnType1	DEPLETED	6.19E-10	0.78	0.02
Clostridium sp L2 50	EnType1	DEPLETED	3.30E-13	0.39	0.00
Coprobacter fastidiosus	EnType1	DEPLETED	2.52E-20	1.17	0.01
Coprococcus sp ART55 1	EnType1	DEPLETED	1.04E-22	1.95	0.02
Deinococcus unclassified	EnType1	DEPLETED	1.49E-11	1.56	0.03

Species	EnteroType	Direction	FDR	Prevalence	Mean Ranked Abundance
Granulicella unclassified	EnType1	DEPLETED	0.0047885	0.00	0.00
Holdemania unclassified	EnType1	DEPLETED	1.19E-11	3.91	0.04
Lachnospiraceae bacterium 1 1 57FAA	EnType1	DEPLETED	9.57E-13	14.06	0.11
Lachnospiraceae bacterium 3 1 46FAA	EnType1	DEPLETED	4.63E-46	8.59	0.06
Lachnospiraceae bacterium 5 1 63FAA	EnType1	DEPLETED	1.65E-58	12.89	0.10
Lachnospiraceae bacterium 7 1 58FAA	EnType1	DEPLETED	3.58E-42	16.02	0.12
Lachnospiraceae bacterium 8 1 57FAA	EnType1	DEPLETED	1.82E-14	11.72	0.09
Megamonas unclassified	EnType1	DEPLETED	0.001888	2.34	0.03
Megasphaera unclassified	EnType1	DEPLETED	5.20E-05	9.77	0.10
Methanobrevibacter unclassified	EnType1	DEPLETED	3.16E-16	0.78	0.01
Mitsuokella unclassified	EnType1	DEPLETED	1.86E-26	8.20	0.10
Olsenella unclassified	EnType1	DEPLETED	1.11E-33	1.95	0.04
Oscillibacter sp KLE 1745	EnType1	DEPLETED	0.0153619	0.39	0.00
Oscillibacter unclassified	EnType1	DEPLETED	7.78E-53	21.88	0.13
Paraprevotella unclassified	EnType1	DEPLETED	1.44E-13	6.25	0.06
Parasutterella excrementihominis	EnType1	DEPLETED	9.55E-20	7.03	0.06
Peptostreptococcaceae noname unclassified	EnType1	DEPLETED	7.87E-26	13.67	0.10
Ruminococcaceae bacterium D16	EnType1	DEPLETED	4.73E-22	3.52	0.04
Ruminococcus sp 5 1 39BFAA	EnType1	DEPLETED	5.07E-50	3.91	0.03
Subdoligranulum unclassified	EnType1	DEPLETED	2.49E-57	61.72	0.19
Treponema succinifaciens	EnType1	DEPLETED	2.63E-08	0.00	0.00
Turicibacter sanguinis	EnType1	DEPLETED	2.03E-09	0.78	0.01
Turicibacter unclassified	EnType1	DEPLETED	3.33E-12	0.39	0.02
Weissella unclassified	EnType1	DEPLETED	0.0145351	1.17	0.01
candidate division TM7 single cell isolate TM7c	EnType1	DEPLETED	0.0064269	0.39	0.01

(B)

Species	EnteroType	Direction	FDR	Prevalence	Mean Ranked Abundance
Faecalibacterium prausnitzii	EnType2	ENRICHED	1.61E-10	98.78	0.52
Eubacterium rectale	EnType2	ENRICHED	5.27E-16	92.88	0.53
Subdoligranulum unclassified	EnType2	ENRICHED	4.07E-58	99.56	0.54
Bifidobacterium adolescentis	EnType2	ENRICHED	1.85E-11	74.08	0.52
Ruminococcus bromii	EnType2	ENRICHED	1.20E-37	73.30	0.50
Bacteroides uniformis	EnType2	ENRICHED	5.71E-109	93.55	0.50
Alistipes putredinis	EnType2	ENRICHED	2.79E-124	79.76	0.48
Bacteroides vulgatus	EnType2	ENRICHED	4.58E-71	88.65	0.51
Ruminococcus sp 5 1 39BFAA	EnType2	ENRICHED	1.92E-76	71.75	0.48
Akkermansia muciniphila	EnType2	ENRICHED	8.98E-29	58.84	0.43
Dialister invisus	EnType2	ENRICHED	7.36E-39	41.60	0.35
Bacteroides dorei	EnType2	ENRICHED	1.77E-70	75.31	0.49
Bacteroides stercoris	EnType2	ENRICHED	2.15E-37	48.83	0.42
Ruminococcus torques	EnType2	ENRICHED	7.25E-29	99.56	0.54
Coprococcus sp ART55 1	EnType2	ENRICHED	4.47E-06	34.04	0.27
Bacteroides ovatus	EnType2	ENRICHED	9.14E-78	86.21	0.49
Eubacterium eligens	EnType2	ENRICHED	3.74E-18	81.65	0.49
Bacteroides caccae	EnType2	ENRICHED	1.87E-66	70.52	0.46
Butyrivibrio crossotus	EnType2	ENRICHED	1.02E-06	18.69	0.21
Parabacteroides merdae	EnType2	ENRICHED	1.65E-65	70.97	0.46
Eubacterium hallii	EnType2	ENRICHED	1.51E-87	90.21	0.58
Barnesiella intestinihominis	EnType2	ENRICHED	1.05E-92	72.53	0.45
Dorea longicatena	EnType2	ENRICHED	5.86E-32	90.66	0.56
Roseburia intestinalis	EnType2	ENRICHED	4.53E-20	74.42	0.46
Alistipes onderdonkii	EnType2	ENRICHED	8.36E-110	76.08	0.48
Eubacterium siraeum	EnType2	ENRICHED	1.39E-26	63.74	0.44
Roseburia inulinivorans	EnType2	ENRICHED	9.19E-20	88.21	0.49
Bacteroides eggerthii	EnType2	ENRICHED	9.19E-26	30.03	0.30
Bacteroides thetaiotaomicron	EnType2	ENRICHED	8.74E-66	75.19	0.47
Coprococcus comes	EnType2	ENRICHED	3.39E-58	85.21	0.55
Bacteroides plebeius	EnType2	ENRICHED	1.08E-22	23.14	0.22
Alistipes shahii	EnType2	ENRICHED	6.88E-84	76.75	0.47
Ruminococcus obeum	EnType2	ENRICHED	1.46E-54	96.77	0.59
Bacteroides cellulosilyticus	EnType2	ENRICHED	6.55E-62	46.83	0.38
Odoribacter splanchnicus	EnType2	ENRICHED	1.29E-77	65.63	0.40

Species	EnteroType	Direction	FDR	Prevalence	Mean Ranked Abundance
Bacteroides coprocola	EnType2	ENRICHED	2.62E-24	21.02	0.22
Dorea formicigenerans	EnType2	ENRICHED	8.20E-20	90.32	0.55
Parabacteroides distasonis	EnType2	ENRICHED	1.11E-58	66.63	0.46
Bacteroidales bacterium ph8	EnType2	ENRICHED	6.66E-93	68.63	0.45
Streptococcus salivarius	EnType2	ENRICHED	8.37E-11	82.42	0.57
Ruminococcus lactaris	EnType2	ENRICHED	1.69E-31	51.95	0.41
Streptococcus thermophilus	EnType2	ENRICHED	2.24E-53	51.84	0.48
Roseburia hominis	EnType2	ENRICHED	2.62E-43	82.20	0.49
Paraprevotella unclassified	EnType2	ENRICHED	3.84E-33	37.26	0.30
Oscillibacter unclassified	EnType2	ENRICHED	7.15E-89	87.88	0.49
Lachnospiraceae bacterium 1 1 57FAA	EnType2	ENRICHED	9.16E-41	45.83	0.36
Eubacterium ventriosum	EnType2	ENRICHED	4.79E-89	63.18	0.48
Alistipes finegoldii	EnType2	ENRICHED	1.35E-74	61.40	0.44
Bifidobacterium catenulatum	EnType2	ENRICHED	1.72E-06	28.48	0.32
Bacteroides intestinalis	EnType2	ENRICHED	1.66E-23	21.91	0.24
Coprococcus catus	EnType2	ENRICHED	3.76E-25	79.76	0.51
Bilophila unclassified	EnType2	ENRICHED	1.34E-37	71.86	0.44
Bacteroides coprophilus	EnType2	ENRICHED	0.0002248	7.12	0.09
Streptococcus parasanguinis	EnType2	ENRICHED	2.20E-13	66.52	0.54
Bacteroides xylanisolvens	EnType2	ENRICHED	2.23E-72	64.40	0.46
Alistipes sp AP11	EnType2	ENRICHED	1.54E-30	25.58	0.21
Eubacterium ramulus	EnType2	ENRICHED	9.38E-36	70.52	0.48
Lachnospiraceae bacterium 5 1 63FAA	EnType2	ENRICHED	1.33E-91	77.53	0.54
Bacteroides finegoldii	EnType2	ENRICHED	1.89E-21	25.14	0.25
Coprococcus eutactus	EnType2	ENRICHED	8.58E-08	11.12	0.16
Lachnospiraceae bacterium 3 1 46FAA	EnType2	ENRICHED	4.34E-88	61.74	0.47
Paraprevotella clara	EnType2	ENRICHED	2.49E-38	31.92	0.27
Clostridium leptum	EnType2	ENRICHED	2.42E-102	57.95	0.48
Clostridium bartlettii	EnType2	ENRICHED	4.23E-11	57.29	0.45
Bifidobacterium animalis	EnType2	ENRICHED	3.74E-18	18.24	0.20
Alistipes indistinctus	EnType2	ENRICHED	2.24E-73	46.38	0.38

Species	EnteroType	Direction	FDR	Prevalence	Mean Ranked Abundance
Bacteroides salyersiae	EnType2	ENRICHED	2.77E-19	17.46	0.18
Anaerostipes hadrus	EnType2	ENRICHED	1.15E-96	69.86	0.53
Alistipes unclassified	EnType2	ENRICHED	2.48E-12	9.90	0.10
Bacteroides clarus	EnType2	ENRICHED	3.89E-32	21.80	0.25
Alistipes sp HGB5	EnType2	ENRICHED	2.97E-05	5.90	0.06
Subdoligranulum sp 4 3 54A2FAA	EnType2	ENRICHED	5.26E-18	19.24	0.17
Parabacteroides johnsonii	EnType2	ENRICHED	3.59E-11	16.46	0.21
Adlercreutzia equolifaciens	EnType2	ENRICHED	1.86E-94	51.61	0.45
Bacteroides sp 2 1 22	EnType2	ENRICHED	0.0018507	7.12	0.07
Lachnospiraceae bacterium 2 1 58FAA	EnType2	ENRICHED	3.63E-12	27.47	0.31
Parasutterella excrementihominis	EnType2	ENRICHED	2.93E-48	33.59	0.35
Lachnospiraceae bacterium 7 1 58FAA	EnType2	ENRICHED	1.20E-57	62.07	0.47
Alistipes senegalensis	EnType2	ENRICHED	2.19E-38	45.72	0.42
Burkholderiales bacterium 1 1 47	EnType2	ENRICHED	4.48E-45	30.14	0.33
Eubacterium cylindroides	EnType2	ENRICHED	4.22E-17	13.24	0.15
Paraprevotella xylaniphila	EnType2	ENRICHED	3.35E-35	23.80	0.26
Lactobacillus delbrueckii	EnType2	ENRICHED	1.04E-18	18.35	0.22
Oscillibacter sp KLE 1745	EnType2	ENRICHED	2.75E-09	9.12	0.08
Anaerotruncus unclassified	EnType2	ENRICHED	5.66E-25	51.72	0.40
Megamonas rupellensis	EnType2	ENRICHED	0.0032595	5.12	0.07
Collinsella intestinalis	EnType2	ENRICHED	0.0309069	4.45	0.05
Coprobacillus sp 29 1	EnType2	ENRICHED	0.036018	3.78	0.04
Coprobacter fastidiosus	EnType2	ENRICHED	6.72E-58	28.92	0.30
Bilophila wadsworthia	EnType2	ENRICHED	1.78E-34	40.49	0.38
Gordonibacter pamelaeae	EnType2	ENRICHED	1.03E-43	28.92	0.33
Holdemania filiformis	EnType2	ENRICHED	8.16E-82	40.16	0.38
Ruminococcaceae bacterium D16	EnType2	ENRICHED	3.48E-58	17.58	0.36
Coriobacteriaceae bacterium phI	EnType2	ENRICHED	5.79E-05	5.90	0.07
Anaerotruncus colihominis	EnType2	ENRICHED	2.74E-40	18.02	0.27

Species	EnteroType	Direction	FDR	Prevalence	Mean Ranked Abundance
Streptococcus mutans	EnType2	ENRICHED	6.96E-06	9.57	0.17
Lactobacillus acidophilus	EnType2	ENRICHED	1.97E-06	8.34	0.09
Eubacterium sp 3 1 31	EnType2	ENRICHED	1.02E-04	8.01	0.10
Lachnospiraceae bacterium 1 4 56FAA	EnType2	ENRICHED	7.89E-23	17.02	0.22
Bacteroides nordii	EnType2	ENRICHED	4.38E-24	13.46	0.20
Propionibacterium freudenreichii	EnType2	ENRICHED	0.0054677	2.89	0.05
Blautia hydrogenotrophica	EnType2	ENRICHED	7.20E-12	7.45	0.12
Clostridiaceae bacterium JC118	EnType2	ENRICHED	3.34E-29	13.57	0.22
Erysipelotrichaceae bacterium 5 2 54FAA	EnType2	ENRICHED	0.0175959	3.67	0.05
Desulfovibrio desulfuricans	EnType2	ENRICHED	9.31E-14	14.02	0.26
Clostridium citroniae	EnType2	ENRICHED	8.06E-33	16.69	0.29
Blautia producta	EnType2	ENRICHED	0.0017104	4.00	0.10
Pseudoflavonifractor capillosus	EnType2	ENRICHED	1.64E-67	18.35	0.33
Holdemania unclassified	EnType2	ENRICHED	7.31E-34	17.35	0.27
Erysipelotrichaceae bacterium 21 3	EnType2	ENRICHED	9.69E-43	11.46	0.34
Leuconostoc mesenteroides	EnType2	ENRICHED	0.0026977	3.78	0.07
Eubacterium dolichum	EnType2	ENRICHED	8.26E-06	4.23	0.06
Butyrivibrio unclassified	EnType2	ENRICHED	1.05E-22	11.35	0.21
Lachnospiraceae bacterium 5 1 57FAA	EnType2	ENRICHED	6.45E-10	6.56	0.12
Subdoligranulum variabile	EnType2	ENRICHED	8.77E-26	7.68	0.36
Clostridium scindens	EnType2	ENRICHED	6.13E-29	2.89	0.21
Ruminococcus albus	EnType2	ENRICHED	6.21E-26	6.34	0.28
Butyricimonas synergistica	EnType2	ENRICHED	1.03E-14	1.89	0.15
Gemella sanguinis	EnType2	ENRICHED	0.0003153	4.45	0.14
Granulicella unclassified	EnType2	ENRICHED	0.0003368	1.11	0.07
Candidatus Zinderia insecticola	EnType2	ENRICHED	4.74E-07	0.56	0.09
Clostridium methylpentosum	EnType2	ENRICHED	0.0024236	1.11	0.04
Bacteroides oleiciplenus	EnType2	ENRICHED	0.0002791	0.44	0.05

Species	EnteroType	Direction	FDR	Prevalence	Mean Ranked Abundance
Acidaminococcus intestini	EnType2	DEPLETED	0.0011979	4.89	0.08
Acinetobacter johnsonii	EnType2	DEPLETED	0.0012132	0.11	0.01
Actinomyces turicensis	EnType2	DEPLETED	0.0004599	2.00	0.04
Actinomyces urogenitalis	EnType2	DEPLETED	0.0002447	0.22	0.00
Aeromonas hydrophila	EnType2	DEPLETED	0.0280664	0.00	0.00
Anaerococcus hydrogenalis	EnType2	DEPLETED	1.67E-05	0.11	0.00
Bacteroides faecis	EnType2	DEPLETED	8.99E-12	26.25	0.33
Bacteroides fragilis	EnType2	DEPLETED	0.0024924	52.50	0.38
Bifidobacterium breve	EnType2	DEPLETED	1.55E-18	7.45	0.10
Bifidobacterium longum	EnType2	DEPLETED	3.07E-14	89.32	0.56
Brachyspira pilosicoli	EnType2	DEPLETED	8.32E-12	0.11	0.00
Catenibacterium mitsuokai	EnType2	DEPLETED	1.72E-26	21.36	0.18
Citrobacter freundii	EnType2	DEPLETED	1.18E-10	5.01	0.07
Clostridium asparagiforme	EnType2	DEPLETED	5.40E-31	18.58	0.27
Clostridium bolteae	EnType2	DEPLETED	2.85E-23	34.71	0.35
Clostridium clostridioforme	EnType2	DEPLETED	0.0001489	12.79	0.16
Clostridium hathewayi	EnType2	DEPLETED	2.16E-19	24.25	0.33
Clostridium innocuum	EnType2	DEPLETED	4.09E-12	6.56	0.24
Clostridium perfringens	EnType2	DEPLETED	0.0029418	6.01	0.09
Clostridium symbiosum	EnType2	DEPLETED	7.10E-13	19.35	0.24
Collinsella aerofaciens	EnType2	DEPLETED	0.0469145	83.54	0.52
Corynebacterium amycolatum	EnType2	DEPLETED	0.013275	0.33	0.01
Eggerthella lenta	EnType2	DEPLETED	4.54E-09	18.69	0.32
Enterobacter cloacae	EnType2	DEPLETED	1.40E-36	9.34	0.12
Enterococcus avium	EnType2	DEPLETED	0.0029192	1.89	0.03
Enterococcus casseliflavus	EnType2	DEPLETED	3.26E-13	0.78	0.01
Enterococcus faecalis	EnType2	DEPLETED	1.01E-22	5.23	0.09
Enterococcus faecium	EnType2	DEPLETED	0.0068564	6.01	0.09
Enterococcus gallinarum	EnType2	DEPLETED	0.0021031	1.11	0.02
Enterococcus hirae	EnType2	DEPLETED	2.81E-07	0.78	0.01
Escherichia coli	EnType2	DEPLETED	6.98E-38	69.86	0.42
Escherichia fergusonii	EnType2	DEPLETED	1.61E-09	0.11	0.00
Eubacterium biforme	EnType2	DEPLETED	0.0106201	39.15	0.30
Finegoldia magna	EnType2	DEPLETED	1.81E-16	1.22	0.03
Flavonifractor plautii	EnType2	DEPLETED	2.31E-37	30.92	0.36
Haemophilus influenzae	EnType2	DEPLETED	0.0406176	0.11	0.00
Klebsiella oxytoca	EnType2	DEPLETED	1.23E-13	3.00	0.05
Klebsiella pneumoniae	EnType2	DEPLETED	3.51E-44	12.57	0.12

Species	EnteroType	Direction	FDR	Prevalence	Mean Ranked Abundance
Lactobacillus iners	EnType2	DEPLETED	0.0429925	0.89	0.01
Lactobacillus rhamnosus	EnType2	DEPLETED	6.61E-12	6.56	0.08
Lactobacillus ruminis	EnType2	DEPLETED	2.72E-13	27.59	0.25
Lactobacillus sakei	EnType2	DEPLETED	4.06E-12	8.34	0.11
Lactococcus garvieae	EnType2	DEPLETED	5.60E-10	1.56	0.03
Leuconostoc lactis	EnType2	DEPLETED	3.83E-26	1.00	0.02
Megasphaera elsdenii	EnType2	DEPLETED	5.42E-08	7.68	0.10
Methanosphaera stadtmanae	EnType2	DEPLETED	1.03E-08	5.45	0.06
Mitsuokella multacida	EnType2	DEPLETED	8.64E-13	8.57	0.08
Mobiluncus curtisii	EnType2	DEPLETED	2.66E-06	0.56	0.01
Parabacteroides goldsteinii	EnType2	DEPLETED	3.21E-14	19.58	0.21
Peptoniphilus harei	EnType2	DEPLETED	4.03E-07	0.67	0.02
Peptoniphilus lacrimalis	EnType2	DEPLETED	1.13E-08	1.00	0.03
Peptoniphilus timonensis	EnType2	DEPLETED	0.0195377	0.22	0.00
Phascolarctobacterium succinatutens	EnType2	DEPLETED	7.27E-08	25.47	0.22
Porphyromonas asaccharolytica	EnType2	DEPLETED	0.0003529	3.89	0.08
Porphyromonas somerae	EnType2	DEPLETED	1.35E-03	1.22	0.03
Prevotella bivia	EnType2	DEPLETED	3.43E-06	2.22	0.05
Prevotella buccae	EnType2	DEPLETED	2.08E-05	1.78	0.04
Prevotella copri	EnType2	DEPLETED	4.83E-06	43.72	0.35
Prevotella disiens	EnType2	DEPLETED	3.70E-03	2.45	0.06
Prevotella stercorea	EnType2	DEPLETED	8.98E-38	14.68	0.15
Prevotella timonensis	EnType2	DEPLETED	9.83E-09	2.22	0.05
Propionibacterium acnes	EnType2	DEPLETED	3.43E-21	0.89	0.02
Propionibacterium avidum	EnType2	DEPLETED	0.0257678	0.00	0.00
Ruminococcus gnavus	EnType2	DEPLETED	0.0017135	47.72	0.39
Staphylococcus epidermidis	EnType2	DEPLETED	9.68E-05	0.00	0.00
Stenotrophomonas maltophilia	EnType2	DEPLETED	2.98E-11	0.56	0.01
Streptococcus infantarius	EnType2	DEPLETED	5.38E-43	1.22	0.03
Streptococcus infantis	EnType2	DEPLETED	0.0281603	8.45	0.25
Streptococcus lutetiensis	EnType2	DEPLETED	8.69E-14	1.67	0.03
Streptococcus peroris	EnType2	DEPLETED	0.0004551	0.44	0.02
Streptococcus sanguinis	EnType2	DEPLETED	0.0001087	11.68	0.27
Streptococcus vestibularis	EnType2	DEPLETED	6.82E-11	22.91	0.30
Shigella sonnei	EnType2	DEPLETED	0.0062806	0.78	0.01

Species	EnteroType	Direction	FDR	Prevalence	Mean Ranked Abundance
Varibaculum cambriense	EnType2	DEPLETED	3.63E-10	0.67	0.02
Veillonella dispar	EnType2	DEPLETED	8.01E-04	16.02	0.28
Veillonella parvula	EnType2	DEPLETED	0.0002866	47.72	0.41
Veillonella ratti	EnType2	DEPLETED	3.61E-06	0.44	0.01
Weissella cibaria	EnType2	DEPLETED	0.008212	1.67	0.03
Weissella confusa	EnType2	DEPLETED	7.96E-35	2.34	0.03
Acinetobacter unclassified	EnType2	DEPLETED	1.78E-08	0.78	0.02
Afipia broomeae	EnType2	DEPLETED	1.06E-11	0.56	0.01
Afipia unclassified	EnType2	DEPLETED	3.77E-13	0.44	0.00
Alloprevotella unclassified	EnType2	DEPLETED	6.43E-18	1.33	0.05
Anaerococcus lactolyticus	EnType2	DEPLETED	2.27E-08	0.11	0.00
Anaerococcus obesiensis	EnType2	DEPLETED	6.56E-11	1.22	0.02
Atopobium vaginae	EnType2	DEPLETED	0.0066578	1.11	0.02
Brachyspira unclassified	EnType2	DEPLETED	1.06E-09	1.56	0.02
Bradyrhizobium sp DFCI 1	EnType2	DEPLETED	4.97E-05	0.44	0.00
Buchnera aphidicola	EnType2	DEPLETED	0.0007542	0.00	0.00
Campylobacter hominis	EnType2	DEPLETED	0.0302258	1.33	0.03
Campylobacter ureolyticus	EnType2	DEPLETED	5.53E-05	0.67	0.01
Citrobacter unclassified	EnType2	DEPLETED	2.07E-16	4.23	0.08
Clostridiales bacterium 1 7 47FAA	EnType2	DEPLETED	3.72E-11	11.23	0.20
Coriobacteriaceae bacterium BV3Ac1	EnType2	DEPLETED	5.22E-05	0.11	0.00
Corynebacterium pyruviciproducens	EnType2	DEPLETED	0.0045958	0.00	0.00
Cupriavidus unclassified	EnType2	DEPLETED	1.28E-07	0.67	0.01
Dialister micraerophilus	EnType2	DEPLETED	1.21E-05	0.11	0.00
Dorea unclassified	EnType2	DEPLETED	0.0127492	8.01	0.08
Eggerthella unclassified	EnType2	DEPLETED	1.42E-08	41.16	0.39
Enhydrobacter aerosaccus	EnType2	DEPLETED	0.0003105	0.11	0.00
Erysipelotrichaceae bacterium 2 2 44A	EnType2	DEPLETED	2.30E-23	13.35	0.33
Erysipelotrichaceae bacterium 6 1 45	EnType2	DEPLETED	4.74E-26	12.01	0.33
Escherichia unclassified	EnType2	DEPLETED	1.85E-25	42.83	0.34
Gardnerella vaginalis	EnType2	DEPLETED	0.0022482	1.89	0.05
Klebsiella unclassified	EnType2	DEPLETED	2.68E-15	5.78	0.09

Species	EnteroType	Direction	FDR	Prevalence	Mean Ranked Abundance
Lachnospiraceae bacterium 3 1 57FAA CT1	EnType2	DEPLETED	1.03E-30	18.91	0.31
Leuconostoc citreum	EnType2	DEPLETED	6.27E-11	3.34	0.06
Leuconostoc unclassified	EnType2	DEPLETED	7.30E-06	0.67	0.02
Megasphaera unclassified	EnType2	DEPLETED	1.88E-08	17.35	0.16
Neisseria flavescens	EnType2	DEPLETED	0.0001004	0.11	0.00
Neisseria meningitidis	EnType2	DEPLETED	0.0182259	0.00	0.00
Neisseria unclassified	EnType2	DEPLETED	1.15E-05	0.56	0.03
Olsenella unclassified	EnType2	DEPLETED	1.30E-08	11.46	0.29
Pantoea unclassified	EnType2	DEPLETED	3.36E-08	5.56	0.07
Peptoniphilus duerdenii	EnType2	DEPLETED	6.71E-05	0.11	0.01
Peptoniphilus rhinitidis	EnType2	DEPLETED	7.53E-04	0.11	0.00
Peptostreptococcaceae noname unclassified	EnType2	DEPLETED	0.0475976	47.61	0.36
Plesiomonas shigelloides	EnType2	DEPLETED	0.0113723	0.00	0.00
Porphyromonas bennonis	EnType2	DEPLETED	7.38E-13	2.11	0.04
Prevotella bergensis	EnType2	DEPLETED	4.62E-05	0.11	0.00
Prevotella buccalis	EnType2	DEPLETED	6.03E-10	1.89	0.04
Prevotella multisaccharivorax	EnType2	DEPLETED	2.76E-11	0.11	0.01
Ralstonia pickettii	EnType2	DEPLETED	0.0190196	0.33	0.00
Ralstonia unclassified	EnType2	DEPLETED	6.24E-10	0.22	0.01
Rhodopseudomonas palustris	EnType2	DEPLETED	2.68E-07	0.44	0.00
Selenomonas bovis	EnType2	DEPLETED	7.00E-18	0.33	0.00
Sphingobacterium unclassified	EnType2	DEPLETED	3.30E-05	0.11	0.00
Stenotrophomonas unclassified	EnType2	DEPLETED	2.30E-07	0.00	0.00
Sutterella wadsworthensis	EnType2	DEPLETED	0.0225672	44.16	0.35
Treponema succinifaciens	EnType2	DEPLETED	1.06E-60	0.11	0.01
Veillonella unclassified	EnType2	DEPLETED	0.0002468	53.62	0.45
Weissella unclassified	EnType2	DEPLETED	1.15E-13	1.78	0.03

(C)

Species	EnteroType	Direction	FDR	Prevalence	Mean Ranked Abundance
Prevotella copri	EnType3	ENRICHED	1.27E-87	100.00	0.79
Faecalibacterium prausnitzii	EnType3	ENRICHED	7.92E-31	100.00	0.69
Eubacterium rectale	EnType3	ENRICHED	8.90E-23	98.47	0.66
Escherichia coli	EnType3	ENRICHED	1.74E-14	94.27	0.66
Prevotella stercorea	EnType3	ENRICHED	1.48E-159	94.66	0.84
Collinsella aerofaciens	EnType3	ENRICHED	3.59E-24	100.00	0.70
Treponema succinifaciens	EnType3	ENRICHED	5.80E-162	41.98	0.58
Butyrivibrio crossotus	EnType3	ENRICHED	7.68E-73	72.90	0.64
Phascolarctobacterium succinatutens	EnType3	ENRICHED	1.25E-70	83.59	0.65
Catenibacterium mitsuokai	EnType3	ENRICHED	4.64E-134	93.51	0.80
Eubacterium biforme	EnType3	ENRICHED	2.13E-73	96.95	0.72
Methanobrevibacter smithii	EnType3	ENRICHED	1.09E-21	64.89	0.51
Eubacterium eligens	EnType3	ENRICHED	2.23E-10	91.60	0.56
Roseburia inulinivorans	EnType3	ENRICHED	1.87E-14	95.80	0.58
Lactobacillus ruminis	EnType3	ENRICHED	5.86E-89	90.08	0.82
Megasphaera unclassified	EnType3	ENRICHED	2.29E-38	53.44	0.49
Escherichia unclassified	EnType3	ENRICHED	1.66E-20	75.95	0.61
Klebsiella pneumoniae	EnType3	ENRICHED	7.20E-48	61.83	0.54
Lactococcus garvieae	EnType3	ENRICHED	3.75E-32	17.18	0.23
Dorea formicigenerans	EnType3	ENRICHED	4.51E-17	98.85	0.65
Clostridium sp L2 50	EnType3	ENRICHED	4.18E-07	32.44	0.27
Peptostreptococcaceae noname unclassified	EnType3	ENRICHED	2.08E-07	59.16	0.46
Streptococcus infantarius	EnType3	ENRICHED	1.54E-103	39.31	0.49
Streptococcus salivarius	EnType3	ENRICHED	0.0176368	82.06	0.59
Enterobacter cloacae	EnType3	ENRICHED	8.78E-61	53.05	0.56
Haemophilus parainfluenzae	EnType3	ENRICHED	1.84E-15	75.19	0.57
Mitsuokella unclassified	EnType3	ENRICHED	6.93E-33	62.98	0.61
Enterococcus hirae	EnType3	ENRICHED	4.74E-24	12.98	0.15
Selenomonas bovis	EnType3	ENRICHED	6.52E-49	14.89	0.20
Methanobrevibacter unclassified	EnType3	ENRICHED	5.12E-29	40.08	0.42
Mitsuokella multacida	EnType3	ENRICHED	6.33E-60	45.04	0.44
Ruminococcus callidus	EnType3	ENRICHED	4.34E-24	75.19	0.58

Species	EnteroType	Direction	FDR	Prevalence	Mean Ranked Abundance
Coprococcus catus	EnType3	ENRICHED	1.90E-08	94.66	0.57
Coprococcus eutactus	EnType3	ENRICHED	5.48E-60	43.89	0.55
Lachnospiraceae bacterium 8 1 57FAA	EnType3	ENRICHED	6.97E-18	63.74	0.46
Clostridium perfringens	EnType3	ENRICHED	1.97E-16	21.37	0.26
Methanosphaera stadtmanae	EnType3	ENRICHED	6.45E-42	31.68	0.33
Desulfovibrio piger	EnType3	ENRICHED	2.63E-17	42.37	0.38
Ruminococcus champanellensis	EnType3	ENRICHED	1.88E-15	16.41	0.18
Streptococcus lutetiensis	EnType3	ENRICHED	8.64E-20	13.74	0.20
Brachyspira pilosicoli	EnType3	ENRICHED	6.87E-33	12.98	0.14
Weissella confusa	EnType3	ENRICHED	5.88E-82	36.64	0.43
Ruminococcus sp JC304	EnType3	ENRICHED	0.00016	10.31	0.10
Rothia mucilaginosa	EnType3	ENRICHED	6.78E-14	30.92	0.48
Lachnospiraceae bacterium 7 1 58FAA	EnType3	ENRICHED	7.52E-08	30.53	0.25
Olsenella unclassified	EnType3	ENRICHED	6.91E-93	67.56	0.78
Leuconostoc lactis	EnType3	ENRICHED	5.89E-61	25.95	0.34
Streptococcus gallolyticus	EnType3	ENRICHED	0.000563	3.44	0.04
Megasphaera elsdenii	EnType3	ENRICHED	1.87E-34	29.39	0.38
Turicibacter unclassified	EnType3	ENRICHED	0.0001004	19.85	0.26
Veillonella dispar	EnType3	ENRICHED	1.55E-08	38.93	0.45
Acidaminococcus fermentans	EnType3	ENRICHED	6.86E-06	12.21	0.16
Streptococcus macedonicus	EnType3	ENRICHED	0.0015849	2.67	0.06
Plesiomonas shigelloides	EnType3	ENRICHED	1.34E-08	2.67	0.04
Oxalobacter formigenes	EnType3	ENRICHED	3.57E-06	30.53	0.32
Escherichia fergusonii	EnType3	ENRICHED	1.19E-27	8.78	0.12
Acidaminococcus intestini	EnType3	ENRICHED	3.26E-13	14.50	0.25
Actinomyces odontolyticus	EnType3	ENRICHED	5.23E-07	22.90	0.37
Brachyspira unclassified	EnType3	ENRICHED	1.21E-35	16.41	0.21
Leuconostoc unclassified	EnType3	ENRICHED	2.81E-22	10.31	0.17
Propionibacterium acnes	EnType3	ENRICHED	5.03E-36	17.94	0.26
Weissella unclassified	EnType3	ENRICHED	2.08E-43	20.23	0.27
Acinetobacter unclassified	EnType3	ENRICHED	2.46E-19	7.63	0.16
Streptococcus gordonii	EnType3	ENRICHED	0.0002342	15.27	0.29
Campylobacter jejuni	EnType3	ENRICHED	1.46E-07	5.34	0.06

Species	EnteroType	Direction	FDR	Prevalence	Mean Ranked Abundance
candidate division TM7 single cell isolate TM7c	EnType3	ENRICHED	2.72E-08	10.31	0.17
Shigella sonnei	EnType3	ENRICHED	3.18E-12	6.11	0.09
Pantoea unclassified	EnType3	ENRICHED	5.69E-05	17.56	0.20
Weissella cibaria	EnType3	ENRICHED	2.75E-14	9.16	0.14
Ruminococcus flavefaciens	EnType3	ENRICHED	4.29E-08	9.92	0.29
Neisseria unclassified	EnType3	ENRICHED	3.92E-11	8.02	0.14
Enterobacter asburiae	EnType3	ENRICHED	1.20E-05	1.15	0.03
Deinococcus unclassified	EnType3	ENRICHED	0.0004814	12.21	0.26
Actinobacillus unclassified	EnType3	ENRICHED	5.21E-05	5.73	0.21
candidate division TM7 single cell isolate TM7b	EnType3	ENRICHED	1.41E-07	4.96	0.09
Alloprevotella unclassified	EnType3	ENRICHED	3.00E-59	14.50	0.37
Clostridium bifermentans	EnType3	ENRICHED	0.0172743	1.91	0.02
Aggregatibacter segnis	EnType3	ENRICHED	0.0003089	4.58	0.09
Eubacterium infirmum	EnType3	ENRICHED	6.71E-06	3.44	0.13
Comamonas unclassified	EnType3	ENRICHED	5.42E-14	6.87	0.19
Neisseria flavescens	EnType3	ENRICHED	5.10E-06	3.05	0.05
Sphingobacterium unclassified	EnType3	ENRICHED	5.18E-11	0.38	0.07
Ralstonia unclassified	EnType3	ENRICHED	6.29E-07	2.29	0.09
Cronobacter sakazakii	EnType3	ENRICHED	2.60E-05	2.29	0.03
Prevotella multisaccharivorax	EnType3	ENRICHED	2.95E-33	1.91	0.15
Aggregatibacter unclassified	EnType3	ENRICHED	2.39E-06	2.29	0.12
Enhydrobacter aerosaccus	EnType3	ENRICHED	0.0003836	1.53	0.04
Aeromonas hydrophila	EnType3	ENRICHED	2.29E-05	1.53	0.03
Actinomyces cardiffensis	EnType3	ENRICHED	1.45E-03	1.53	0.03
Halomonas unclassified	EnType3	ENRICHED	1.27E-03	1.53	0.04
Propionibacterium propionicum	EnType3	ENRICHED	6.01E-03	1.15	0.05
Klebsiella variicola	EnType3	ENRICHED	8.89E-08	0.00	0.06
Enterobacter mori	EnType3	ENRICHED	1.75E-02	0.38	0.02
Buchnera aphidicola	EnType3	ENRICHED	3.42E-12	0.00	0.06
Neisseria meningitidis	EnType3	ENRICHED	3.05E-05	0.00	0.05

Species	EnteroType	Direction	FDR	Prevalence	Mean Ranked Abundance
Limnohabitans unclassified	EnType3	ENRICHED	0.0011355	0.38	0.02
Prevotella melaninogenica	EnType3	ENRICHED	2.11E-04	0.00	0.03
Acinetobacter johnsonii	EnType3	DEPLETED	6.79E-04	1.53	0.06
Akkermansia muciniphila	EnType3	DEPLETED	4.67E-34	17.56	0.11
Alistipes finegoldii	EnType3	DEPLETED	2.79E-20	20.99	0.14
Alistipes indistinctus	EnType3	DEPLETED	1.92E-25	4.96	0.04
Alistipes onderdonkii	EnType3	DEPLETED	2.02E-56	7.25	0.05
Alistipes putredinis	EnType3	DEPLETED	5.18E-41	25.95	0.10
Alistipes shahii	EnType3	DEPLETED	4.03E-09	47.71	0.23
Anaerostipes hadrus	EnType3	DEPLETED	1.33E-25	23.66	0.18
Anaerotruncus colihominis	EnType3	DEPLETED	1.33E-18	0.00	0.01
Bacteroides caccae	EnType3	DEPLETED	2.22E-25	26.72	0.16
Bacteroides cellulosilyticus	EnType3	DEPLETED	1.01E-21	6.49	0.07
Bacteroides clarus	EnType3	DEPLETED	4.37E-12	0.00	0.02
Bacteroides coprocola	EnType3	DEPLETED	1.06E-08	0.76	0.03
Bacteroides dorei	EnType3	DEPLETED	5.41E-40	25.95	0.14
Bacteroides eggerthii	EnType3	DEPLETED	3.27E-09	7.63	0.09
Bacteroides finegoldii	EnType3	DEPLETED	0.0016548	5.73	0.10
Bacteroides fragilis	EnType3	DEPLETED	5.07E-08	31.68	0.23
Bacteroides intestinalis	EnType3	DEPLETED	9.08E-05	3.82	0.08
Bacteroides massiliensis	EnType3	DEPLETED	2.67E-15	8.40	0.08
Bacteroides nordii	EnType3	DEPLETED	8.26E-08	1.15	0.03
Bacteroides ovatus	EnType3	DEPLETED	1.27E-33	45.42	0.18
Bacteroides plebeius	EnType3	DEPLETED	5.99E-07	4.96	0.04
Bacteroides salyersiae	EnType3	DEPLETED	1.09E-09	0.00	0.00
Bacteroides stercoris	EnType3	DEPLETED	9.99E-08	22.90	0.21
Bacteroides thetaiotaomicron	EnType3	DEPLETED	3.27E-30	24.81	0.15
Bacteroides uniformis	EnType3	DEPLETED	1.82E-66	42.75	0.11
Bacteroides vulgatus	EnType3	DEPLETED	9.91E-39	59.92	0.19
Bacteroides xylanisolvens	EnType3	DEPLETED	2.07E-15	23.66	0.19
Barnesiella intestinihominis	EnType3	DEPLETED	1.23E-23	27.48	0.14
Bifidobacterium animalis	EnType3	DEPLETED	1.84E-10	0.76	0.01
Bifidobacterium bifidum	EnType3	DEPLETED	3.94E-18	12.21	0.11
Bifidobacterium breve	EnType3	DEPLETED	7.70E-07	4.20	0.05
Bifidobacterium longum	EnType3	DEPLETED	1.27E-51	44.27	0.21
Bifidobacterium pseudocatenulatum	EnType3	DEPLETED	0.0004512	37.02	0.39

Species	EnteroType	Direction	FDR	Prevalence	Mean Ranked Abundance
Blautia hydrogenotrophica	EnType3	DEPLETED	6.00E-06	0.00	0.00
Blautia producta	EnType3	DEPLETED	3.59E-05	0.00	0.00
Butyricimonas synergistica	EnType3	DEPLETED	0.0236959	1.15	0.04
Cetobacterium somerae	EnType3	DEPLETED	5.93E-05	2.29	0.05
Citrobacter freundii	EnType3	DEPLETED	4.00E-06	7.25	0.22
Clostridium asparagiforme	EnType3	DEPLETED	1.01E-19	0.00	0.01
Clostridium bolteae	EnType3	DEPLETED	1.84E-33	2.67	0.04
Clostridium citroniae	EnType3	DEPLETED	1.53E-13	1.53	0.06
Clostridium clostridioforme	EnType3	DEPLETED	1.43E-11	0.00	0.00
Clostridium hathewayi	EnType3	DEPLETED	6.86E-28	3.05	0.04
Clostridium innocuum	EnType3	DEPLETED	1.68E-13	0.00	0.02
Clostridium leptum	EnType3	DEPLETED	1.01E-39	4.96	0.06
Clostridium ramosum	EnType3	DEPLETED	4.22E-11	1.53	0.02
Clostridium scindens	EnType3	DEPLETED	3.93E-11	0.00	0.01
Clostridium symbiosum	EnType3	DEPLETED	1.75E-18	1.15	0.01
Dialister invisus	EnType3	DEPLETED	2.32E-29	2.67	0.02
Dialister succinatiphilus	EnType3	DEPLETED	2.11E-23	2.67	0.30
Dorea longicatena	EnType3	DEPLETED	0.0018576	97.71	0.57
Eggerthella lenta	EnType3	DEPLETED	3.13E-26	1.15	0.04
Enterococcus faecalis	EnType3	DEPLETED	0.0003415	3.82	0.06
Eubacterium cylindroides	EnType3	DEPLETED	1.52E-07	0.00	0.00
Eubacterium dolichum	EnType3	DEPLETED	0.0461139	0.00	0.00
Eubacterium hallii	EnType3	DEPLETED	0.0086039	79.77	0.37
Eubacterium ventriosum	EnType3	DEPLETED	4.22E-16	22.14	0.16
Finegoldia magna	EnType3	DEPLETED	0.0090816	0.76	0.01
Flavonifractor plautii	EnType3	DEPLETED	4.95E-33	3.05	0.04
Gordonibacter pamelaeae	EnType3	DEPLETED	4.57E-23	1.53	0.02
Holdemania filiformis	EnType3	DEPLETED	2.80E-34	1.15	0.02
Lactobacillus acidophilus	EnType3	DEPLETED	0.0020036	0.00	0.00
Lactobacillus delbrueckii	EnType3	DEPLETED	2.06E-06	3.44	0.04
Lactobacillus gasseri	EnType3	DEPLETED	1.16E-08	1.15	0.03
Lactobacillus mucosae	EnType3	DEPLETED	2.46E-10	17.94	0.24
Lactobacillus rhamnosus	EnType3	DEPLETED	2.65E-10	0.00	0.00
Lactobacillus sakei	EnType3	DEPLETED	0.0001218	0.00	0.00
Odoribacter splanchnicus	EnType3	DEPLETED	3.69E-17	24.81	0.11
Parabacteroides distasonis	EnType3	DEPLETED	4.54E-18	31.68	0.19

Species	EnteroType	Direction	FDR	Prevalence	Mean Ranked Abundance
Parabacteroides goldsteinii	EnType3	DEPLETED	0.0110673	5.34	0.08
Parabacteroides merdae	EnType3	DEPLETED	1.84E-17	36.26	0.18
Paraprevotella clara	EnType3	DEPLETED	4.23E-14	1.91	0.02
Paraprevotella xylaniphila	EnType3	DEPLETED	1.23E-11	2.29	0.03
Prevotella buccae	EnType3	DEPLETED	5.01E-15	0.00	0.18
Pseudoflavonifractor capillosus	EnType3	DEPLETED	7.09E-27	0.00	0.00
Ruminococcus gnavus	EnType3	DEPLETED	1.42E-31	12.60	0.11
Streptococcus anginosus	EnType3	DEPLETED	0.0094841	17.94	0.32
Streptococcus thermophilus	EnType3	DEPLETED	3.45E-32	4.58	0.08
Acidaminococcus unclassified	EnType3	DEPLETED	0.0288259	1.53	0.02
Adlercreutzia equolifaciens	EnType3	DEPLETED	5.29E-37	3.05	0.03
Alistipes sp AP11	EnType3	DEPLETED	1.98E-11	0.76	0.01
Alistipes unclassified	EnType3	DEPLETED	6.95E-19	24.05	0.33
Anaerostipes unclassified	EnType3	DEPLETED	0.0144028	0.00	0.00
Bacteroidales bacterium ph8	EnType3	DEPLETED	5.29E-24	17.18	0.12
Burkholderiales bacterium 1 1 47	EnType3	DEPLETED	8.17E-17	2.67	0.06
Butyrivibrio unclassified	EnType3	DEPLETED	6.20E-08	1.15	0.03
Citrobacter unclassified	EnType3	DEPLETED	2.86E-12	7.63	0.28
Clostridiaceae bacterium JC118	EnType3	DEPLETED	1.99E-11	0.76	0.01
Clostridiales bacterium 1 7 47FAA	EnType3	DEPLETED	6.74E-15	0.00	0.00
Coprobacillus unclassified	EnType3	DEPLETED	2.39E-17	3.05	0.05
Coprobacter fastidiosus	EnType3	DEPLETED	1.98E-23	0.00	0.00
Coriobacteriaceae bacterium phI	EnType3	DEPLETED	0.0163971	0.00	0.00
Eggerthella unclassified	EnType3	DEPLETED	5.96E-51	2.67	0.02
Erysipelotrichaceae bacterium 21 3	EnType3	DEPLETED	1.14E-27	0.00	0.01

Species	EnteroType	Direction	FDR	Prevalence	Mean Ranked Abundance
Erysipelotrichaceae bacterium 2 2 44A	EnType3	DEPLETED	2.39E-25	0.38	0.02
Erysipelotrichaceae bacterium 3 1 53	EnType3	DEPLETED	0.0355428	0.00	0.00
Erysipelotrichaceae bacterium 6 1 45	EnType3	DEPLETED	1.06E-28	0.00	0.00
Eubacterium sp 3 1 31	EnType3	DEPLETED	9.31E-05	0.00	0.00
Holdemania unclassified	EnType3	DEPLETED	2.37E-13	0.00	0.03
Lachnospiraceae bacterium 1 1 57FAA	EnType3	DEPLETED	8.51E-18	10.69	0.08
Lachnospiraceae bacterium 1 4 56FAA	EnType3	DEPLETED	4.54E-14	0.00	0.00
Lachnospiraceae bacterium 2 1 58FAA	EnType3	DEPLETED	1.93E-08	8.78	0.12
Lachnospiraceae bacterium 3 1 46FAA	EnType3	DEPLETED	3.81E-23	17.94	0.14
Lachnospiraceae bacterium 3 1 57FAA CT1	EnType3	DEPLETED	5.59E-25	0.38	0.01
Lachnospiraceae bacterium 5 1 57FAA	EnType3	DEPLETED	1.20E-05	0.00	0.00
Lachnospiraceae bacterium 5 1 63FAA	EnType3	DEPLETED	1.96E-17	31.68	0.23
Lachnospiraceae bacterium 9 1 43BFAA	EnType3	DEPLETED	0.0134536	0.00	0.01
Lactobacillus casei paracasei	EnType3	DEPLETED	1.01E-11	3.05	0.04
Leuconostoc citreum	EnType3	DEPLETED	3.88E-29	21.37	0.30
Odoribacter unclassified	EnType3	DEPLETED	0.0226007	12.21	0.17
Oscillibacter sp KLE 1745	EnType3	DEPLETED	0.0034102	0.00	0.00
Oscillibacter unclassified	EnType3	DEPLETED	3.66E-19	62.60	0.20
Paraprevotella unclassified	EnType3	DEPLETED	6.26E-11	8.02	0.08
Parasutterella excrementihominis	EnType3	DEPLETED	1.53E-16	3.82	0.07
Ruminococcaceae bacterium D16	EnType3	DEPLETED	6.85E-22	0.00	0.04
Ruminococcus sp 5 1 39BFAA	EnType3	DEPLETED	1.03E-13	33.21	0.18
Subdoligranulum sp 4 3 54A2FAA	EnType3	DEPLETED	1.45E-08	0.38	0.00
Subdoligranulum unclassified	EnType3	DEPLETED	0.010663	100.00	0.37
Veillonella unclassified	EnType3	DEPLETED	2.39E-14	21.30	0.04

FootNote: 1. Ranked Abundances were calculated by ranking the relative abundances obtained for a given species across all samples, in such a manner that the sample with the highest relative abundance gets a value of 1 and that with the lowest abundance gets a value of 0. This approach ensures that the relative abundances of all species are at the same range (0 to 1) (irrespective of differences in the range of actual relative abundances). 2. Prevalence indicates the percentage of samples in a given Enterotype that contain the species with a relative abundance of greater than 0.01
