# Supplementary material for: Metagenomic analysis reveals distinct patterns of gut lactobacillus prevalence, abundance, and geographical variation in health and disease
Source: Gut Microbes. 2020 Sep 28;12(1):1822729. doi: 10.1080/19490976.2020.1822729 (PMC7524322; doi:10.1080/19490976.2020.1822729)
Supplement: Supplemental Material [file KGMI_A_1822729_SM9159.zip › Supplementary information/Revised_SupplementaryTableS1.pdf]

Supplementary Table S1: Details of the shotgun metagenomic datasets included in the current analysis, with distribution of geography, age, gender, BMI and study conditions (control, diseased, different treatment) in each dataset along with the total number of samples

Dataset Name	Geography		Age Group					BMI			Gender	
	Country	Region	Infant	Children/Teen	Young	Middle	Elderly	Min	Median	Max	Male	Female
AsnicarF_2017	ITA	EU	8	0	0	0	0	NA	NA	NA	7	9
Bengtsson-PalmeJ_2015	SWE	EU	0	0	70	0	0	NA	NA	NA	18	52
BritoIL_2016	FJI	Others	7	54	55	43	13	NA	NA	NA	58	114
FengQ_2015	AUT	EU	0	0	0	26	128	18.0	27.8	43.6	87	67
HMP_2012	USA	North America	0	12	135	0	0	NA	NA	NA	82	65
HanniganGD_2017	CAN, USA	North America	0	0	0	0	0	NA	NA	NA	NA	NA
KarlssonFH_2013	SWE, DEU, FRA, NOR, DNK, SVK, HUN, EST, FIN, ISL	EU	0	0	0	0	145	NA	NA	NA	NA	145
KosticAD_2015	FIN, EST	EU	118	2	0	0	0	NA	NA	NA	53	67
LeChatelierE_2013	DNK	EU	0	0	0	0	0	18.1	30.7	46.6	NA	NA
LiJ_2014	CHN, DNK, ESP	Asia, EU	0	0	0	0	0	NA	NA	NA	NA	NA
LiJ_2017	CHN	Asia	0	0	0	0	0	NA	NA	NA	NA	NA
LiSS_2016	NLD	EU	0	0	0	0	0	NA	NA	NA	55	NA

Dataset Name	Number of Samples			Non Control Conditions
	Total	Controls	Non Controls	
AsnicarF_2017	16	16	0	
Bengtsson-PalmeJ_2015	70	70	0	
BritoIL_2016	172	172	0	
FengQ_2015	154	61	93	CRC, adenoma
HMP_2012	147	147	0	
HanniganGD_2017	82	28	54	adenoma, CRC
KarlssonFH_2013	145	43	102	IGT, T2D
KosticAD_2015	124	89	31	T1D
LeChatelierE_2013	292	292	0	
LiJ_2014	260	260	0	
LiJ_2017	196	41	99	hypertension
LiSS_2016	55	5	50	metabolic_syndrome, FMT

Dataset Name	Geography		Age Group					BMI			Gender	
	Country	Region	Infant	Children/Teen	Young	Middle	Elderly	Min	Median	Max	Male	Female
LiuW_2016	MNG	Asia	0	0	0	0	0	NA	NA	NA	NA	NA
LomanNJ_2013	DEU	EU	0	0	0	0	0	NA	NA	NA	NA	NA
LoombaR_2017	USA	North America	0	0	0	0	0	NA	NA	NA	NA	NA
LouisS_2016	DEU	EU	0	0	0	0	0	NA	NA	NA	NA	NA
NielsenHB_2014	DNK, ESP	EU	0	9	90	237	58	16.9	25.6	42.0	NA	NA
Obregon-TitoAJ_2015	PER, USA	Others, North America	4	20	27	6	0	14.1	21.5	31.5	27	30
OlmMR_2017	USA	North America	37	0	0	0	0	NA	NA	NA	NA	NA
PasolliE_2018	MDG	Others	0	1	68	26	2	16.9	20.9	29.5	51	46
QinJ_2012	CHN	Asia	0	3	103	175	63	15.6	23.8	37.5	190	154
QinN_2014	CHN	Asia	0	1	72	139	25	15.9	22.0	35.2	156	81
RampelliS_2015	TZA, ITA	Others, EU	0	6	26	4	2	NA	NA	NA	22	16
RaymondF_2016	CAN	North America	0	0	72	0	0	19.0	23.9	29.8	33	39
SchirmerM_2016	NLD	EU	0	107	301	31	26	15.1	22.3	34.4	200	265
SmitsSA_2017	TZA	Others	0	8	10	6	0	NA	NA	NA	15	12
ThomasAM_2018a	ITA	EU	0	0	0	20	60	19	25	37	52	28
VatanenT_2016	RUS, EST, FIN	EU	762	23	0	0	0	NA	NA	NA	449	336
VincentC_2016	CAN	North America	0	0	0	0	229	NA	NA	NA	124	105
VogtmannE_2016	USA	North America	0	0	7	36	61	16.8	24.1	38.371	74	30

Dataset Name	Number of Samples			Non Control Conditions
	Total	Controls	Non Controls	
LiuW_2016	110	110	0	
LomanNJ_2013	43	0	43	STEC
LoombaR_2017	86	0	86	fatty_liver
LouisS_2016	92	92	0	
NielsenHB_2014	396	248	148	IBD
Obregon-TitoAJ_2015	58	58	0	
OlmMR_2017	37	0	37	premature_born
PasolliE_2018	112	112	0	
QinJ_2012	363	174	170	T2D
QinN_2014	237	114	123	cirrhosis
RampelliS_2015	38	38	0	
RaymondF_2016	72	36	36	cephalosporins
SchirmerM_2016	471	471	0	
SmitsSA_2017	40	40	0	
ThomasAM_2018a	80	24	56	adenoma, CRC
VatanenT_2016	785	614	171	multiple diseases *
VincentC_2016	229	196	33	CDI
VogtmannE_2016	110	52	52	CRC

Dataset Name	Geography		Age Group					BMI			Gender	
	Country	Region	Infant	Children/Teen	Young	Middle	Elderly	Min	Median	Max	Male	Female
WenC_2017	CHN	Asia	0	4	69	23	1	16.9	20.5	33.2	56	41
XieH_2016	GBR	EU	0	0	4	94	152	16	24	44	NA	250
YuJ_2015	CHN	Asia	0	0	0	0	0	NA	NA	NA	NA	NA
ZellerG_2014	FRA, DEU	EU	0	0	11	62	126	15	25	40	117	82
FranzosaCA_2018	USA	North America	0	1	116	55	47	NA	NA	NA	NA	NA
ELDERMET	IRE	EU	0	0	0	0	189	12.1	27	47.7	NA	NA

Dataset Name	Number of Samples			Non Control Conditions
	Total	Controls	Non Controls	
WenC_2017	97	0	97	AS
XieH_2016	250	250	0	
YuJ_2015	128	53	75	CRC
ZellerG_2014	199	66	133	CRC, adenoma
FranzosaCA_2018	219	56	163	IBD
ELDERMET	189	189	0	

Notes: *: multiple diseases include bronchitis, otitis, respiratoryinf, tonsillitis, stomatitis, infectiousgastroenteritis, salmonellosis, skininf, pneumonia, sepsis, fever, NK, cystitis, cough, pyelonephritis, pyelonefritis, suspinf

Non-control disease conditions may include diseases as well as different treatments (like antibiotic or FMT). The latter groups were not considered at all

Out of the 36 datasets, 5 cohorts contained no or less than 5 control samples and were not considered in the LbTyping taking the number of datatests considered in the LbTyping to 31.
