# Supplementary material for: Metagenomic analysis reveals distinct patterns of gut lactobacillus prevalence, abundance, and geographical variation in health and disease
Source: Gut Microbes. 2020 Sep 28;12(1):1822729. doi: 10.1080/19490976.2020.1822729 (PMC7524322; doi:10.1080/19490976.2020.1822729)
Supplement: Supplemental Material [file KGMI_A_1822729_SM9159.zip › Supplementary information/Revised_SupplementaryTableS2.pdf]

Supplementary Table S2: (A) Results of Linear Regression based analysis quantfying the association of Lactobacilli detection rates (number of species detected per sample) with different sample-specific host demographic (like country, region, age, BMI and gender) after taking into account the study-specific effects as confounder. As noted in [47], these are core set of metadata that are available for at least 30% of individuals in the curatedMetagenomic Data repository

Factor	F-value	P-value
**Country**	**8.26**	**2.20E-16**
**Age-group**	**7.74**	**1.30E-08**
**Study-Condition**	**8.19**	**2.20E-16**
BMI	2.14	0.14
Gender	0.83	0.58
Antibiotic Use	2.19	0.12

For each factor X, Linear Regression Analysis was performed by comparing different linear regression models:
Model 1: Detection Rate ~ as.factor(Study_Name)
Model 2: Detection Rate ~ as.factor(Study_Name) + X
Comparing Model 1 and Mode 2 using Log-Ratio Likelihood test
LRT(Model 1, Model 2)

(B) Results of PERMANOVA analysis showing the association of the different metadata as above with the abundance profile of different Lactobacilli after taking int account the study-specific technical factors factors like sequencing depth, DNA extraction method and median read length reflective of sequencing technology as confounders

Factor	R-squared	P-value
**Country**	**0.115**	**1.00E-03**
**Age-group**	**0.054**	**1.00E-03**
**Study-Condition**	**0.078**	**1.00E-03**
BMI	0.006	0.54
Gender	0.002	0.45

For each factor X, PERMANOVA was performed using the adonis function of the vegan package of R:
adonis(SpearmanDistance Matrix of Lactobacillus Species Abundance Profiles ~ ExtractionMethod + SequenceDepth+Read Length+X
