# Supplementary material for: Metagenomic analysis reveals distinct patterns of gut lactobacillus prevalence, abundance, and geographical variation in health and disease
Source: Gut Microbes. 2020 Sep 28;12(1):1822729. doi: 10.1080/19490976.2020.1822729 (PMC7524322; doi:10.1080/19490976.2020.1822729)
Supplement: Supplemental Material [file KGMI_A_1822729_SM9159.zip › Supplementary information/Revised_SupplementaryTableS3.pdf]

Supplementary Table S1. Number of samples from apparently healthy controls in which Lactobacillus species was detected with abundance > 0.01% along with their prevalence pattern.

Species	Number of Samples in which detected with abundance > 0.01	Type
Lactobacillus_ruminis	505	
Lactobacillus_casei_paracasei	243	
Lactobacillus_gasseri	196	
Lactobacillus_delbrueckii	188	
Lactobacillus_rhamnosus	179	
Lactobacillus_salivarius	147	
Lactobacillus_fermentum	139	
Lactobacillus_mucosae	124	High
Lactobacillus_acidophilus	91	
Lactobacillus_sakei	79	
Lactobacillus_plantarum	70	Med
Lactobacillus_crispatus	36	
Lactobacillus_iners	30	
Lactobacillus_vaginalis	27	
Lactobacillus_oris	27	
Lactobacillus_amylovorus	26	
Lactobacillus_curvatus	25	
Lactobacillus_johnsonii	19	
Lactobacillus_reuteri	18	
Lactobacillus_brevis	17	
Lactobacillus_helveticus	12	
Lactobacillus_animalis	10	
Lactobacillus_sanfranciscensis	9	
Lactobacillus_buchneri	9	
Lactobacillus_otakiensis	6	
Lactobacillus_jensenii	5	Low
Lactobacillus_saerimneri	4	
Lactobacillus_versmoldensis	3	
Lactobacillus_parafarraginis	3	
Lactobacillus_zeae	2	
Lactobacillus_ultunensis	2	
Lactobacillus_hilgardii	2	
Lactobacillus_gastricus	2	
Lactobacillus_antri	2	
Lactobacillus_sp_7_1_47FAA	1	
Lactobacillus_rossiae	1	
Lactobacillus_pentosus	1	
Lactobacillus_kefiranofaciens	1	
Lactobacillus_fructivorans	1	
Lactobacillus_farciminis	1	
Lactobacillus_equicursoris	1	
Lactobacillus_coryniformis	1	
Lactobacillus_shenzhenensis	1	
Lactobacillus_murinus	1	

Lactobacillus_ingluviei	1	
Lactobacillus_coleohominis	1	
Lactobacillus_amylolyticus	1	Rare
