# Supplementary material for: Metagenomic analysis reveals distinct patterns of gut lactobacillus prevalence, abundance, and geographical variation in health and disease
Source: Gut Microbes. 2020 Sep 28;12(1):1822729. doi: 10.1080/19490976.2020.1822729 (PMC7524322; doi:10.1080/19490976.2020.1822729)
Supplement: Supplemental Material [file KGMI_A_1822729_SM9159.zip › Supplementary information/Revised_SupplementaryTableS5.pdf]

Supplementary Table S5: Logistic regression analysis showing the association of Enterotypes with different (A) Age-groups (B) Regions and (C) LbTypes

(A) Age Class (adjusting for region)

Age Class*	Enterotype 1		Enterotype 2		Enterotype 3	
	Estimate	P-value	Estimate	P-value	Estimate	P-value
Infants	5.34	<2.2e-16	-4.9	<2.2e-16	-1.15	0.23
ChildrenTeenager	-1.95	4.60E-04	0.85	0.025	0.56	0.42
Young	-4.64	<2.2e-16	2.47	<2.2e-16	-0.05	0.91
Middle	-2.94	5.20E-11	1.43	4.17E-07	0.61	0.35
Elderly	1.72	2.44E-06	1.76	7.90E-16	-1.46	0.15

* indicates that for each Age Class X and Enterotype Y, the logistic regression models were implemented as: glm((age class X = 1 and not X = 0) ~ factor(region) + (Enterotype Y = 1 and not Y = 0)). The model computes the association between each enterotype and age-class after accounting for the biases in the representation of each age-class across various regions.

(B) Region (adjusting for age)

Region#	Enterotype 1		Enterotype 2		Enterotype 3	
	Estimate	P-value	Estimate	P-value	Estimate	P-value
North America	3.96	< 2.2e-16	-2.67	<2.2e-16	-16.35	2.50E-05
Europe	0.01	9.50E-01	3.32	<2.2e-16	6.67	<2.2e-16
Asia	-2.14	3.50E-05	2.7	8.75E-11	-2.63	3.12E-05
Others	-17.9	<2.2e-16	-3.79	<2.2e-16	8.47	<2.2e-16

* indicates that for each Region A and Enterotype Y, the logistic regression models were implemented as: glm((Region A = 1 and not A = 0) ~ age + (Enterotype Y = 1 and not Y = 0)). The model computes the association between each enterotype and region after accounting for the variations in the age of the individuals belonging to the various regions.

(B) LbType (adjusting for region and age)

LbTypes	Enterotype 1		Enterotype 2		Enterotype 3	
	Estimate	P-value	Estimate	P-value	Estimate	P-value
LbType 1	1.9	1.17E-08	-1.75	5.10E-08	-1.3	5.30E-01
LbType 2	1	1.20E-05	-0.96	2.11E-05	-0.75	6.30E-01
LbType 3	-0.01	9.30E-01	0.08	6.30E-01	-1.08	1.15E-01
LbType 4	-3.52	2.69E-08	3.24	5.02E-15	-0.91	6.50E-01
LbType 5	-1.42	5.73E-08	1	5.41E-04	1.1	6.20E-02
LbType 6	0.38	5.30E-01	0.13	8.20E-01	1.02	0.054
