# Supplementary material for: Metagenomic analysis reveals distinct patterns of gut lactobacillus prevalence, abundance, and geographical variation in health and disease
Source: Gut Microbes. 2020 Sep 28;12(1):1822729. doi: 10.1080/19490976.2020.1822729 (PMC7524322; doi:10.1080/19490976.2020.1822729)
Supplement: Supplemental Material [file KGMI_A_1822729_SM9159.zip › Supplementary information/Revised_SupplementaryTableS6.pdf]

Supplementary Table S6: (A) Results of the Fisher Test Analysis of the association of the continental regions and the LbTypes showing (A) The enrichment estimate and (B) the FDR of the Fisher Test P-value (Corrected using Benjamini Hochberg)

Estimate	Asia	EU	NorthAmerica	Others
Gasseri_Salivarius_Fermentum (GSF)	-0.39	0.23	0.49	-1.51
Casei	-0.91	0.84	-0.17	-1.40
Mixed	0.07	0.12	0.40	-0.70
Delbrueckii	-0.97	0.77	-0.09	-1.46
Ruminis	0.39	-0.74	-0.82	1.30
Ruminis_Delbrueckii (RD)	0.52	-0.26	-1.51	0.18

BH-Corrected FDR (Fishers' Test P)	Asia	EU	NorthAmerica	Others
Gasseri_Salivarius_Fermentum (GSF)	**2.76E-02**	**2.14E-02**	**1.75E-05**	**1.24E-10**
Casei	**1.61E-07**	**3.40E-22**	3.92E-01	**4.60E-15**
Mixed	3.68E-01	**5.63E-02**	**7.45E-06**	**2.21E-15**
Delbrueckii	**4.92E-05**	**1.04E-12**	6.35E-01	**1.57E-09**
Ruminis	**7.53E-07**	**9.87E-46**	**9.89E-11**	**5.11E-75**
Ruminis_Delbrueckii (RD)	**3.12E-03**	**5.63E-02**	**8.62E-02**	3.02E-01
