# Supplementary material for: Metagenomic analysis reveals distinct patterns of gut lactobacillus prevalence, abundance, and geographical variation in health and disease
Source: Gut Microbes. 2020 Sep 28;12(1):1822729. doi: 10.1080/19490976.2020.1822729 (PMC7524322; doi:10.1080/19490976.2020.1822729)
Supplement: Supplemental Material [file KGMI_A_1822729_SM9159.zip › Supplementary information/Revised_SupplementaryTableS7.pdf]

Supplementary Table S7: Results of the region-stratified Logistic Regression Analysis associating age, gender and BMI with the prevalence rate of Lactobacilli. Logistic regressions were performed separately within each geographical region (after accounting for intra-region country-specific variations). Non diseased samples within each region were collated and subsequently Logistic regresions were performed as glm(LactobacillusDetected (Yes=1 and No=1) ~ Country + (Age or BMI or Gender), method="binomial"). Method="binomial" indicates that it is a logistic regression. Estimate gives the extent and direction and P gives the significance (or strength) of the association. ** indicates P < 0.01, * indicates P < 0.05, bold indicates P < 0.1

(A) Europe	Estimate	P	
**Age**	**0.231**	**0.010**	*
**BMI**	**0.024**	**0.054**	
Gender	-0.146	0.194	

(B) North America	Estimate	P	
**Age**	**0.98**	**0.00**	**
BMI	0.10	0.12	
Gender	-0.29	0.21	

(C) Asia	Estimate	P
Age	0.03	0.89
BMI	-0.05	0.26
Gender	0.00	0.99

(D) Other	Estimate	P
**Age**	**-1.32**	**0.10**
BMI	0.00	0.97
Gender	0.00	0.97
