# Supplementary material for: Metagenomic analysis reveals distinct patterns of gut lactobacillus prevalence, abundance, and geographical variation in health and disease
Source: Gut Microbes. 2020 Sep 28;12(1):1822729. doi: 10.1080/19490976.2020.1822729 (PMC7524322; doi:10.1080/19490976.2020.1822729)
Supplement: Supplemental Material [file KGMI_A_1822729_SM9159.zip › Supplementary information/SupplementaryFiguresDoc.pdf]

SUPPLEMENTARY FIGURES

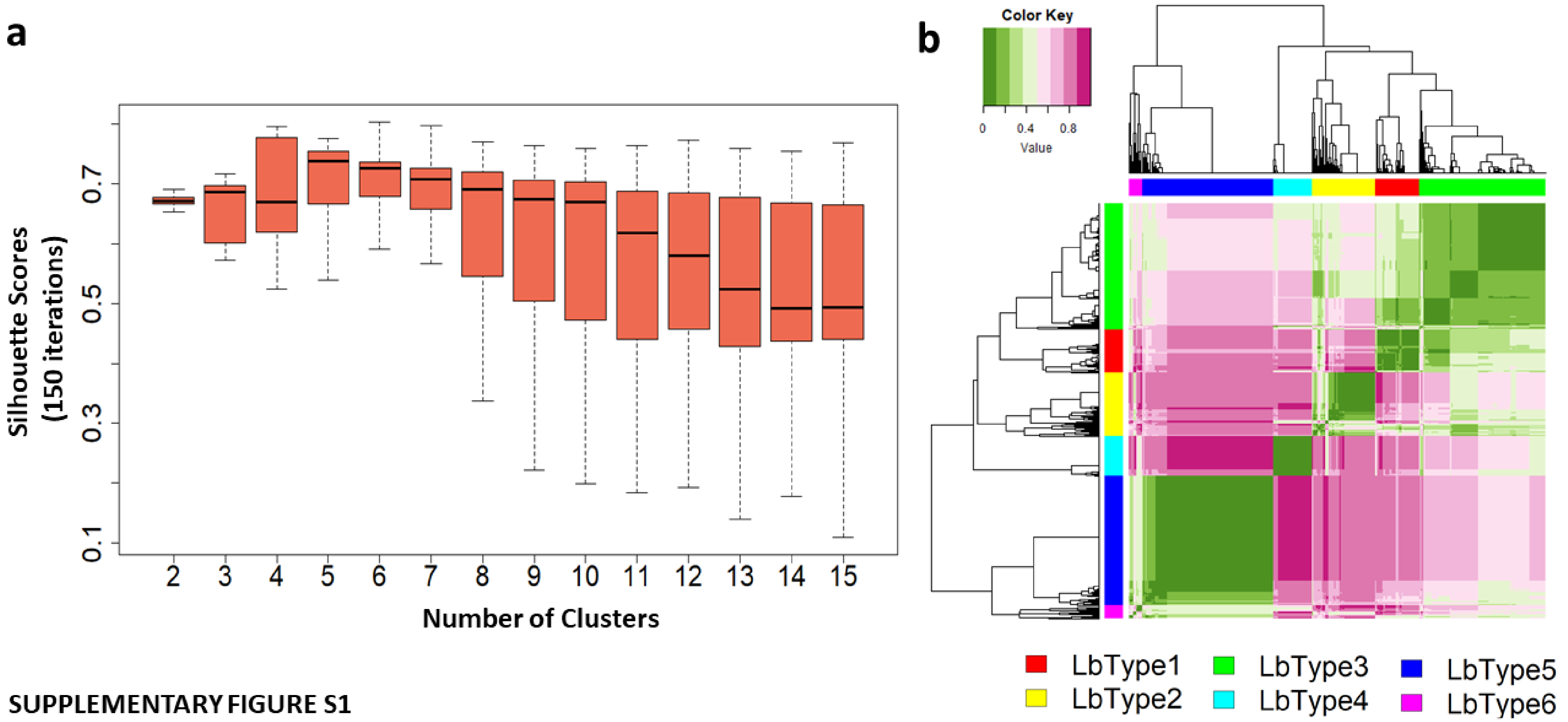

SUPPLEMENTARY FIGURE S1

Supplementary Figure S1. a. Boxplots showing the distribution of Silhouette Scores obtained for various cluster 'k' numbers across the 150 iterations performed for identifying an optimal number of LbTypes. b. Heatmap showing the intra-sample Euclidean distances along with the samples belonging to the various Lactobacillotypes demarcated along the major sub-branches of the sample relatedness trees (both along the rows and columns). The sample-relatedness tree were obtained using Ward-D2 clustering of the Euclidean distances between the top three PCoA Axes (obtained using Spearman distances between the Lactobacilli composition profile). Samples belonging to the different Lactobacillotypes are indicated in different colors.

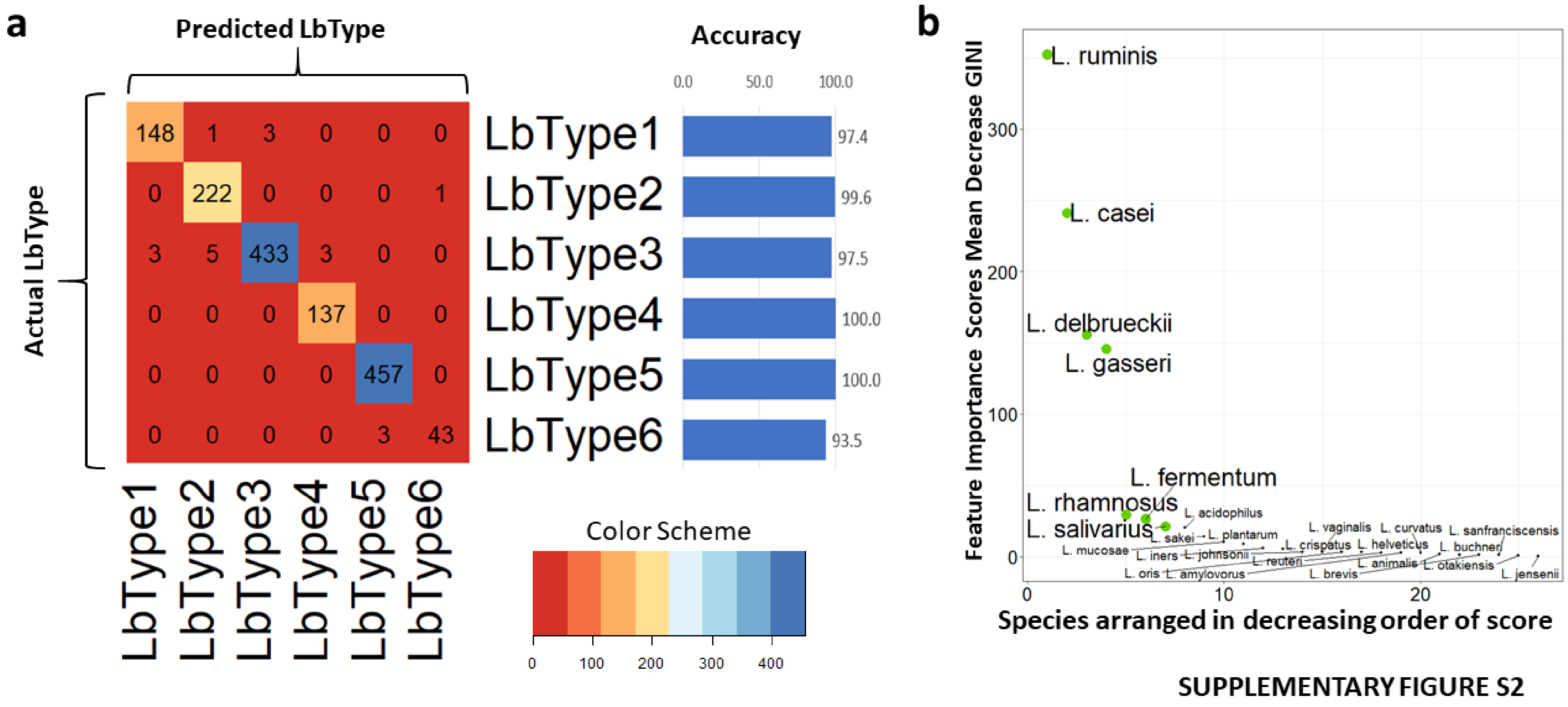

Supplementary Figure S2. a. Confusion Matrix showing the Random Forest model performance on the classification of LbTypes based on Lactobacillus species profiles (using the 100 iterations of the two-fold cross validation scheme). Each cell is coloured based on its corresponding value as indicated by the Coloring Scheme in the bottom right hand corner. b. Plots showing the Feature importance scores of the various species. Species are arranged on the X-axis in decreasing order of their feature importance scores and the feature importance scores themselves are plotted on the Y-axis. Species corresponding to each point label are indicated. Species with Feature importance scores of greater than 20 are shown in green points with larger font.

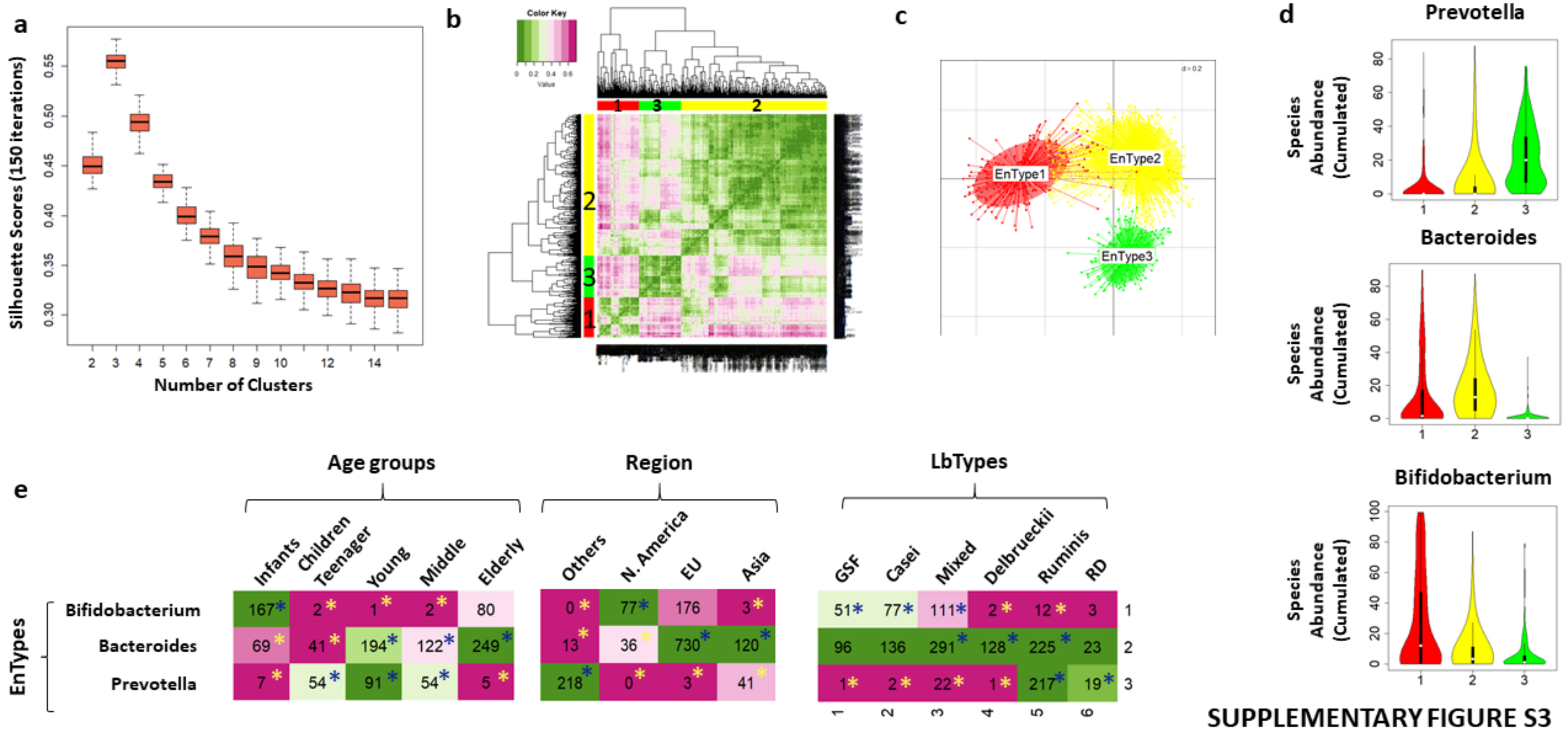

Supplementary Figure S3. a. Boxplots showing the distribution of Silhouette Scores obtained for various cluster ‘k’ numbers across the 150 iterations performed for identifying an optimal number of Enterotypes. b. Heatmap showing the intra-sample Euclidean distances along with the samples belonging to the various Enterotypes demarcated along the three major sub-branches of the sample relatedness trees (both along the rows and columns). The sample-relatedness tree was obtained using Ward-D2 clustering of the Euclidean distances between the top three PCoA (Principal Coordinate Analysis) Axes (obtained using Spearman distances between the full gut microbiome taxa composition profile). Samples belonging to the different Enterotypes are indicated in different colors.The PCoA plot itself is shown in **(c). d.** Boxplot showing the abundance distributions of the three main driver taxa corresponding to the three Enterotypes. **e.** Heatmap showing the detection of frequency of each Enterotypes in individuals belonging to each Age-group, region as well as within six different LbTypes. Association of the different Enterotypes with the different age-groups were computed using Fishers’ exact tests. *s in blue and yellow indicate significant positive or negative association with Benjamini-Hochberg FDR < 0.1, respectively.

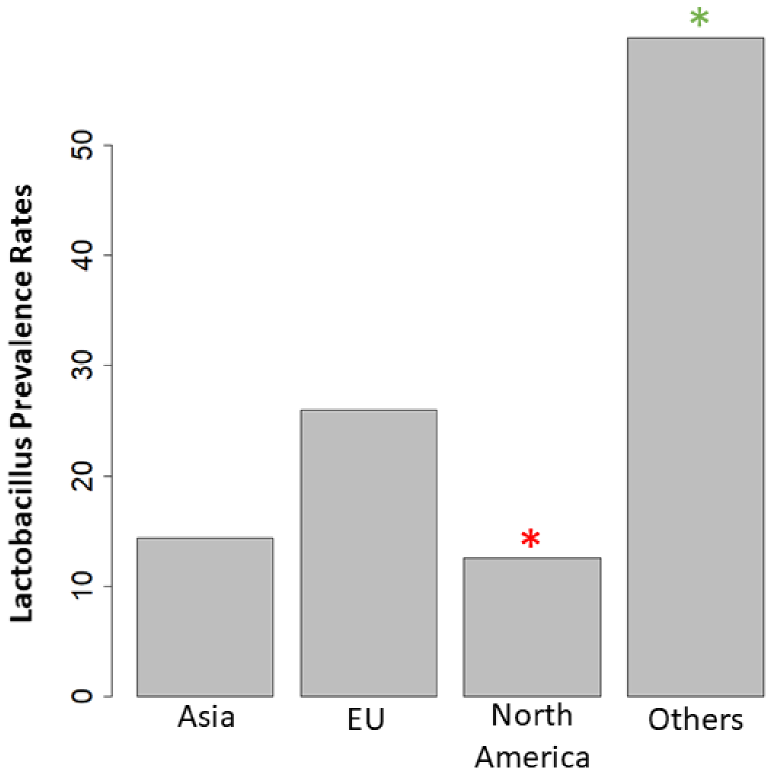

SUPPLEMENTARY FIGURE S4

Supplementary Figure S4. Prevalence rates of Lactobacilli across different regions. *s in orange and green indicate significantly high or low prevalence rates, respectively. The significance of the associations was computed using logistic regression models that computed the association between detection rate and region after adjusting for study-specific effects as confounder (See Methods).

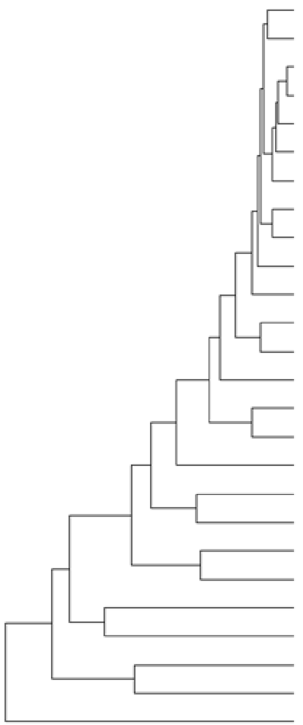

	CAN	USA	AUT	DEU	DNK	ESP	EST	FIN	FRA	GBR	ITA	NLD	RUS	SWE	IRE	CHN	MNG	FJI	PER	MDG	TZA
L. johnsonii	4	1	1	0	0	1	2	0	1	0	1	0	2	0	0	6	0	0	0	0	0
L. reuteri	2	1	2	0	5	2	0	2	2	0	0	1	1	0	0	0	0	0	0	0	0
L. buchneri	0	0	1	0	0	1	0	0	0	0	0	0	5	0	0	0	0	0	0	2	0
L. otakiensis	0	0	1	0	0	0	0	0	0	0	0	1	4	0	0	0	0	0	0	0	0
L. helveticus	1	0	1	0	0	0	2	0	0	1	0	0	0	0	0	0	7	0	0	0	0
L. jensenii	4	0	0	0	0	0	0	0	0	0	0	0	0	0	0	0	0	1	0	0	0
L. animalis	2	1	2	0	0	0	0	0	0	0	0	0	0	0	0	0	0	0	0	4	1
L. oris	1	0	2	0	1	2	4	0	0	3	0	0	7	0	0	2	5	0	0	0	0
L. brevis	1	0	2	0	0	1	0	0	0	1	0	0	12	0	0	0	0	0	0	0	0
L. sanfranciscensis	1	0	3	1	0	0	0	0	1	0	0	1	0	0	0	2	0	0	0	0	0
L. curvatus	5	0	2	0	1	2	1	0	1	1	2	5	5	0	0	0	0	0	0	0	0
L. amylovorus	1	0	5	0	2	8	0	0	0	3	0	1	0	0	0	2	0	1	1	2	0
L. vaginalis	2	0	1	0	3	6	1	0	1	4	1	0	0	0	0	3	3	2	0	0	0
L. plantarum	4	1	5	1	1	4	1	2	3	1	0	5	29	5	0	1	1	1	0	5	0
L. crispatus	10	2	1	0	1	6	0	0	1	3	0	2	0	0	0	6	0	2	0	1	1
L. iners	19	2	0	0	0	2	0	0	0	2	0	0	0	1	0	1	0	3	0	0	0
L. acidophilus	0	1	3	1	7	1	9	13	1	21	0	23	5	5	0	1	0	0	0	0	0
L. mucosae	0	3	4	0	12	8	3	0	5	4	0	8	1	3	0	22	10	11	4	24	2
L. sakei	2	3	3	0	5	15	0	1	9	1	0	34	1	3	0	1	1	0	0	0	0
L. fermentum	15	4	9	0	4	5	6	1	3	8	2	10	12	0	35	9	6	1	5	3	1
L. salivarius	18	2	5	0	15	6	0	0	4	3	0	6	1	0	28	35	8	5	0	10	1
L. gasseri	29	5	12	0	7	7	11	4	9	20	7	6	28	0	39	8	0	0	0	3	0
L. rhamnosus	13	7	11	0	0	2	25	46	2	4	2	12	53	2	0	0	0	0	0	0	0
L. casei	10	5	11	4	12	15	15	24	11	23	2	17	61	7	13	5	0	0	0	8	0
L. delbrueckii	6	8	15	3	6	16	3	1	7	19	2	66	1	19	0	3	11	1	0	0	1
L. ruminis	3	6	1	2	100	8	7	0	8	13	0	28	21	8	0	15	80	105	24	66	10

SUPPLEMENTARY FIGURE S5

Supplementary Figure S5. Heatmap showing the frequency of detection of different Lactobacilli across the different nationalities. Values presented are the number of subjects in the respective nationalities in which the indicated lactobacillus species was detected at an abundance threshold of 0.01%.
